# Effects of Exercise Rehabilitation on Cardiorespiratory Fitness in Long-COVID-19 Survivors: A Meta-Analysis

**DOI:** 10.3390/jcm13123621

**Published:** 2024-06-20

**Authors:** Sothida Nantakool, Piangkwan Sa-nguanmoo, Supatcha Konghakote, Busaba Chuatrakoon

**Affiliations:** 1 Environmental-Occupational Health Sciences and Non Communicable Diseases Research Center, Research Institute for Health Sciences, Chiang Mai University, Chiang Mai 50200, Thailand; sothida.n@cmu.ac.th; 2 Department of Physical Therapy, Faculty of Associated Medical Sciences, Chiang Mai University, Chiang Mai 50200, Thailand; piangkwan.s@cmu.ac.th (P.S.-n.); supatcha.k@cmu.ac.th (S.K.)

**Keywords:** exercise rehabilitation, long-COVID-19 survivors, cardiorespiratory fitness, peak oxygen consumption, six-minute walk test

## Abstract

**Background**: Poor cardiorespiratory fitness poses the highest risk of mortality. Long-COVID-19 survivors exhibit a reduced cardiorespiratory fitness (CRF). While exercise rehabilitation, such as cardiopulmonary exercise, is used for long-COVID-19 survivors, the effects of exercise on CRF in this population remain inconclusive. In this study, we aim to systematically summarise and synthesise whether exercise rehabilitation improves CRF among long-COVID-19 survivors. **Methods**: A comprehensive search was performed through PubMed, CINAHL, Embase, Scopus, and the Cochrane Library (since their inception to November 2023) and study reference lists. Studies presenting the effects of exercise rehabilitation on CRF (peak oxygen consumption (VO_2peak_) and six-minute walk distance (6MWD)) in long-COVID-19 survivors were identified. The standardised mean difference (SMD), mean difference (MD), and 95% confidence interval (CI) were used for analyses. The certainty of evidence was measured using a Grading of Recommendation Assessment, Development and Evaluation approach. **Results**: Twelve eligible studies (five RCTs and seven non-RCTs) with 682 participants were analysed. The meta-analysis showed significantly improved 6MWDs (MD 76.47, 95% CI 59.19–93.71, low certainty) and significantly greater 6MWDs (SMD 0.85, 95% CI 0.11–1.59, very low certainty) in the exercise rehabilitation group compared to the control group. A significantly improved 6MWD was found in subgroups of young to middle-aged adults and subgroups of patients who undertook aerobic exercise combined with resistance and respiratory exercise and centre-based training programs. **Conclusions**: Exercise rehabilitation is effective for improving CRF, as measured by the 6MWD in long-COVID-19 survivors. Improvements are likely to be more pronounced in specific subgroups of young to middle-aged adults and patients undertaking aerobic exercise combined with resistance and respiratory exercise and centre-based training programs. However, recommendations for clinical practice are limited due to the very low evidence certainty.

## 1. Introduction

Currently, the coronavirus disease 2019 (COVID-19) situation is stable; reductions in new cases of 77% and new deaths of 88% have been reported since the beginning of 2021 [1]. Despite the huge reduction in its impact, impairment following the recovery stage is possible. Some COVID-19 survivors exhibit long-term persistent symptoms [2,3]. The U.S. Centers for Disease Control and Prevention (CDC) has defined the wide range of long-term health consequences that occur four or more weeks after the onset of COVID-19 as post-COVID-19 or long-COVID-19 conditions [4]. Breathlessness has been reported as the most common long-COVID-19 symptom [5,6]. COVID-19 not only attacks the respiratory system in the long term but also impairs other systems, such as the cardiovascular and musculoskeletal systems [7,8].

Cardiorespiratory fitness (CRF) is a marker indicating the combined ability of the respiratory, cardiovascular, and musculoskeletal systems to transport oxygen to skeletal muscle mitochondria during physical activity [9]. As a result of multiorgan impairment following SARS-CoV-2 infection, long-COVID-19 survivors are likely to exhibit a lower CRF; extensive research has revealed a reduced CRF in long-COVID-19 survivors when compared to those without COVID-19 [10,11,12,13,14]. Long-COVID-19 survivors of an advanced age and with severe symptoms are more impacted, as it has been reported that they exhibit reductions in CRF of 29% and 47%, respectively [13,14]. A low CRF is linked to a poor quality of life [15] and is the highest risk factor for a reduced life expectancy [16]. Thus, helping patients survive COVID-19 is not the ultimate goal of public health; identifying suitable patient management is more challenging and is required to improve the CRF of long-COVID-19 survivors.

Exercise, primarily focused on cardiopulmonary exercise, is recommended as a cornerstone of rehabilitation for individuals suffering from chronic respiratory and cardiovascular diseases [17,18]. In addition to improving symptoms, such exercise further benefits cardiorespiratory fitness, morbidity, and mortality [19]. With respect to COVID-19, exercise rehabilitation has been applied to long-COVID-19 survivors [20,21,22,23]. Several systematic reviews and meta-analyses have attempted to provide synthesised evidence on the beneficial effects of exercise rehabilitation on various health aspects (e.g., anxiety, depression, dyspnoea, and physical function) in those with COVID [24,25,26,27,28]. However, the shortcomings of these studies are that they (i) focus on health aspects without considering cardiorespiratory fitness [25,27] and (ii) report evidence from combined acute and long-COVID-19 participants [24,25,26,27,28]. It remains unknown whether exercise rehabilitation provides a positive effect on cardiorespiratory fitness in post-COVID-19 survivors. Recently, a growing body of evidence has highlighted the effects of exercise rehabilitation on cardiorespiratory fitness via the peak oxygen consumption and the six-minute walk distance measured in long-COVID-19 survivors. Some individual findings revealed the favourable consequences of such exercises on the CRF among these patients [20,22,23], but some evidence failed to support the use of exercise rehabilitation to improve the CRF in this patient group [21,29]. To clarify this, we aim to systematically summarise and synthesise whether exercise rehabilitation improves the CRF among long-COVID-19 survivors. In the current study, we expect to provide scientific evidence as a part of guidelines for patient care.

## 2. Materials and Methods

### 2.1. Search Strategy

This review followed the Preferred Reporting Items for Systematic Reviews and Meta-Analyses (PRISMA) guidelines [30]. We conducted a literature search of five electronic databases, PubMed (1996-November 2023), CINAHL (1961-November 2023), Scopus (2004-November 2023), and Embase (1980-November 2023), and the Cochrane Library (1993-November 2023), as well as their reference lists to identify all relevant articles. Two search terms were used: COVID (“COVID” OR “coronavirus”) and cardiorespiratory fitness (“cardiorespiratory fitness” OR “physical fitness” OR “cardiopulmonary fitness” OR “fitness performance” OR “cardiorespiratory performance”) (Supplementary Table S1). The review protocol was registered in the PROSPERO database (CRD42023393318).

### 2.2. Study Selection

Eligible articles were selected based on a PICOS approach: (i) participants aged ≥18 years old with a long-COVID-19 condition according to the definition of the Centers for Disease Control and Prevention (CDC) (the wide range of long-term health consequences which occur four or more weeks after the onset of COVID-19) [4], (ii) interventional articles determining the effects of any type of exercise rehabilitation, with no restriction on exercise approaches (i.e., centre-based exercise, home-based exercise, and telerehabilitation), (iii) outcomes of interest indicating cardiorespiratory fitness (i.e., peak oxygen consumption (VO_2peak_) and the six-minute walking distance (6MWD)), (iv) experimental research design encompassing randomised controlled trials (RCTs) and non-RCTs, (v) all languages, and (vi) both published and unpublished articles. Articles meeting the following criteria were excluded: studies investigating animal models, studies without an available full text, and studies containing incomplete data for analyses. The retrieved articles were initially screened by two independent reviewers (SN and PS) based on the determined inclusion and exclusion criteria. The same reviewers then performed a full-text screening to record the final eligible articles. Disagreement after inter-reviewer discussion was resolved by a third reviewer (BC).

### 2.3. Risk of Bias Assessment

Two independent reviewers (SN and PS) were responsible for the methodological quality assessment. The revised Cochrane risk of bias tool for randomised trials (RoB2) covering the following five domains was used to evaluate RCTs’ risk of bias [31]: (i) risk of bias arising from the randomisation process, (ii) risk of bias due to deviations from intended interventions (effect of assignment and adhering to intervention), (iii) risk of bias due to missing outcome data, (iv) risk of bias in measurement of the outcome, and (v) risk of bias in the selection of the reported results. Each domain was scored as “low risk of bias”, “some concerns”, or “high risk of bias”. The sum of all domains of the RoB2 was interpreted as “low risk of bias”, “some concerns”, or “high risk of bias”. The risk of bias in non-randomised studies of interventions (ROBINS-I) was used to appraise the non-randomised controlled trials’ risk of bias. This tool contains seven domains: (i) bias due to confounding, (ii) bias in the selection of study participants, (iii) bias in classification of intervention, (iv) bias due to deviations from intended interventions, (v) bias due to missing data, (vi) bias in measurement outcomes, and (vii) bias in the selection of the reported results [32]. Each domain was judged as “low risk of bias”, “moderate risk of bias”, “serious risk of bias”, “critical risk of bias”, or “no information”. The risk of bias for each study was classified into five categories: low risk of bias, moderate risk of bias, serious risk of bias, critical risk of bias, and no information. In the case of any disagreement between the reviewers, the decision was made by a third reviewer (BC).

### 2.4. Certainty of Evidence

The Grading of Recommendation Assessment, Development and Evaluation (GRADE) approach was used to evaluate the overall certainty for the body of evidence [33]. GRADE can evaluate results as having a high certainty, moderate certainty, low certainty, and very low certainty of evidence based on the consideration of the following domains: risk of bias, inconsistency, indirectness, imprecision, and publication bias [34]. Two independent reviewers (SN and PS) were assigned to evaluate these evidence certainties. A consensus was reached between the two reviewers if there was any disagreement.

### 2.5. Data Extraction

Two reviewers (BC and SK) independently extracted the following article details: author, publication year, study design, participant characteristics (i.e., age, gender, and comorbidities), details of COVID-19 (i.e., hospitalisation duration and long-COVID-19 duration), intervention details (i.e., type of exercise, frequency of exercise, duration of exercise, and exercise approaches), CRF outcome (i.e., VO_2peak_ and 6MWD), and its result. For two-arm studies, characteristics of the intervention and control groups were extracted. For single-arm studies, only the characteristics of the intervention group were recorded. Incomplete outcome data were obtained by sending an email to the study’s authors. If there was no response within a week, the relative incomplete article was excluded. There was a discussion between the two reviewers and a consensus was reached if there was any disagreement.

### 2.6. Data Synthesis

All statistical analyses were conducted using STATA Statistical Software, version 17 (StataCorp LLP, College Station, TX, USA). For RCTs, the standardised mean difference (SMD) and 95% confidence interval (CI) were calculated using the sample number and pre and post changes in mean and standard deviation (SD) in the intervention and control groups. In case there were no pre or post changes in mean and SD, these data were computed based on the following formulas [35]:

Meanchange = Meanfinal − Meanbaseline(1)

SDchange = square root (SD^2^baseline + SD^2^final − (2 × r × SDbaseline × SDfinal)) (2)

r, the pre-post change correlation coefficient, was conservatively estimated as 0.7 [36]. Effect sizes in RCTs were indicated by SMD and were defined as large (0.8), moderate (0.5), or small (0.2) [37].

For non-RCTs, pooled meta-analyses of single-group studies were performed using mean ± SD changes pre and post intervention. The DerSimonian–Laird method with the random-effects model was used as the main method for all meta-analyses. Heterogeneity was investigated using a *p*-value of Cochrane’s Q < 0.1, together with the degree of I^2^. I^2^ values of <25%, 25–75%, and >75% indicate a low, moderate, and high heterogeneity, respectively [38]. In the case of heterogeneity, we attempted to identify its possible source. A subgroup analysis was performed based on the mean-aged population, the type of exercise, and exercise rehabilitation approaches. A sensitivity analysis was conducted by removing most studies affecting the influence plot. Publication bias was considered using a visual funnel plot, a contour-enhanced funnel plot, Begg’s test, and Egger’s test.

## 3. Results

### 3.1. Search Results

**Figure 1:**
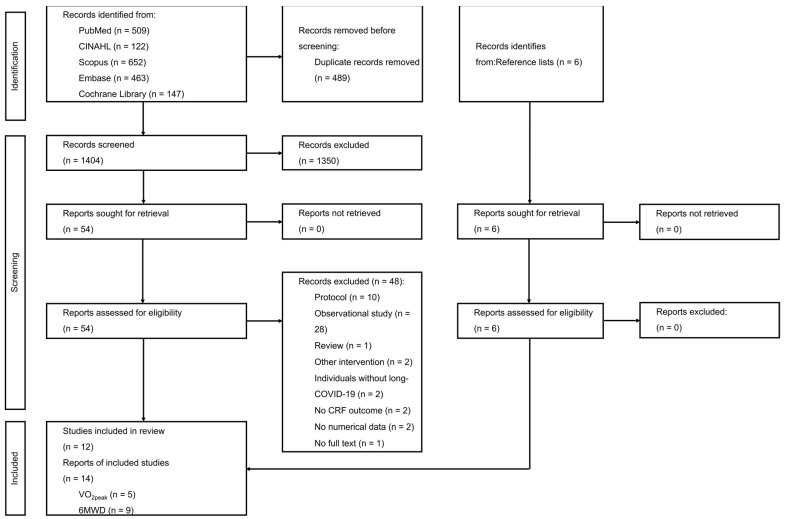
Preferred Reporting Items for Systematic Reviews and Meta-Analyses 2020 diagram.

A PRISMA diagram showing the summarised review process is illustrated in Figure 1. A total of 1893 studies were initially identified and retrieved from five electronic databases. After excluding duplicate studies, the titles and abstracts of 1404 studies were screened. Of these, 54 studies were eligible for full-text assessment. Forty-eight studies were excluded from this review due to using other research designs (*n* = 39), performing other interventions (*n* = 2), investigating other populations (*n* = 2), having no available outcomes relevant to cardiorespiratory fitness (*n* = 2), not providing key numerical data for meta-analysis (*n* = 2), and having no full text available (*n* = 1). Only six studies remained that were eligible for meta-analysis. Six studies from reference lists were additionally included in this review. Finally, 12 studies with 14 reports were included for meta-analysis (i.e., 5 reports documenting VO_2peak_ and 9 reports documenting 6MWD) (Supplementary Table S2).

### 3.2. Characteristics of Included Studies

The characteristics of the included studies are detailed in Table 1. The eligible studies consisted of five RCTs [21,29,39,40,41] and seven non-RCTs [20,22,23,42,43,44,45]. All non-RCTs had a single-group study design which contained pre- and post-intervention data. All studies involved a total of 682 participants, with a mean age of 46.3 years old, and a higher number of males (53.7% male, 46.3% female). Three comorbidities, hypertension, diabetes, and dyslipidaemia, were extracted. Nine out of twelve studies reported these comorbidities [21,22,23,39,40,41,43,44,45]. Among these studies, hypertension was the major comorbidity (38%), followed by dyslipidaemia (30.8%) and diabetes (18.3%). Eight studies reported the hospitalisation duration, widely ranging from 6 to 38.3 days [20,21,22,40,42,43,45]. All included studies documented the long-COVID-19 condition duration, which ranged from 5 to 44.3 weeks. Most studies were conducted in Europe [22,23,29,42,44,45], followed by Asia [20,40,43] and South America [21,39,41].

The following section details the exercise rehabilitation programs. All eligible studies studied combined exercises. Of these, six studies used an aerobic exercise plus resistance exercise program [21,23,39,41,42,44], five studies used aerobic exercise combined with resistance and respiratory exercise programs [22,29,40,43,45], and only one study utilised an aerobic exercise plus respiratory exercise program [20]. The duration of exercise sessions varied from 20 to 100 min, with the most common exercise frequency as 3 to 5 days a week and wide ranges of exercise program durations (3 to 16 weeks). Regarding exercise rehabilitation approaches, most studies were designed to deliver exercise rehabilitation in centre-based training [20,23,29,42,43,44], while the remaining studies used telerehabilitation training [21,22,39,40] and home-based training [41,45].

For the outcome of cardiorespiratory fitness, five studies reported VO_2peak_ values [23,29,41,42,44] as a CRF outcome, whereas the other nine studies reported 6MWD values [20,21,22,39,40,42,43,44,45].

### 3.3. Effect of Exercise Rehabilitation on Cardiorespiratory Fitness

**Figure 2:**
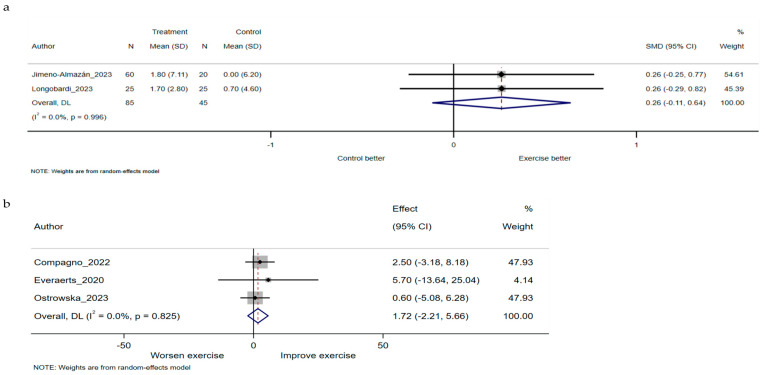
SMD and mean difference are calculated from the random-effects model. (**a**) Meta-analysis of randomised controlled trials for VO_2peak_ between exercise rehabilitation group and control group. (**b**) Meta-analysis of non-randomised controlled trials for VO_2peak_ between exercise rehabilitation group and control group. CI, confidence interval; Effect, mean difference between pre and post intervention; N, number of participants; SD, standard deviation; SMD, standardised mean difference; VO_2peak_, peak oxygen consumption [23,29,41,42,44].

VO_2peak_ and 6MWD values were reported as outcomes representing cardiorespiratory fitness. There were four main analyses: RCTs for VO_2peak_ and 6MWD, and non-RCTs for VO_2peak_ and 6MWD. Pooled effect estimates showed that exercise rehabilitation had a comparable effect on VO_2peak_ compared to the control program (SMD 0.26, 95% CI −0.11 to 0.64, I^2^ 0%, two studies) (Figure 2a). There was no effect of pre-post intervention changes on VO_2peak_ (mean difference 1.72, 95% CI −2.21 to 5.66, I^2^ 0%, three studies) (Figure 2b).

**Figure 3:**
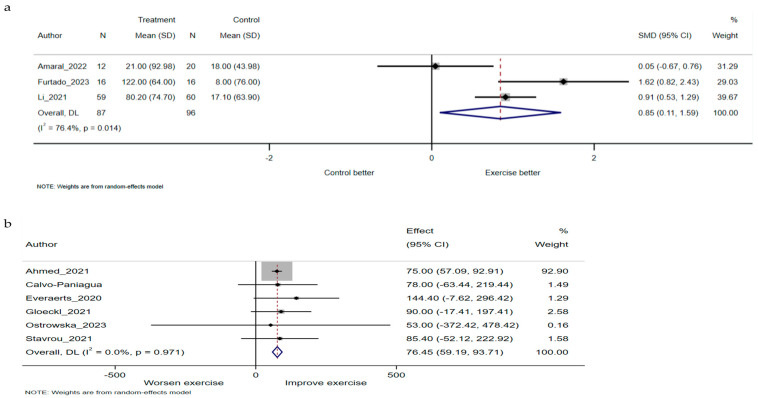
SMD and mean difference are calculated from the random-effects model. (**a**) Meta-analysis of randomised controlled trials for 6MWD between exercise rehabilitation group and control group. (**b**) Meta-analysis of non-randomised controlled trials for 6MWD between exercise rehabilitation group and control group. CI, confidence interval; Effect, mean difference between pre and post intervention; N, number of participants; SD, standard deviation; SMD, standardised mean difference; 6MWD, six-minute walk distance [20,21,22,39,40,42,43,44,45].

Regarding 6MWD, meta-analysis showed that participants in the exercise rehabilitation group had a significantly longer 6MWD than those in the control group (SMD 0.85, 95% CI 0.11 to 1.59, I^2^ 76.4%, three studies) (Figure 3a), and they exhibited a significant improvement in the 6MWD of 76.46 m after completing exercise rehabilitation (mean difference 76.46, 95% CI 59.19 to 93.71, I^2^ 0%, six studies) (Figure 3b).

Subgroup analyses of cardiorespiratory outcomes (i.e., VO_2peak_ and 6MWD) were performed based on three possible sources: (i) mean age of the population (middle-aged adults versus older-aged adults), (ii) type of exercise (aerobic plus breathing exercise, aerobic plus resistance exercise, aerobic exercise combined with resistance and respiratory exercise), and (iii) exercise rehabilitation approaches (centre-based training, telerehabilitation, home-based training).

Subgroup analyses of non-RCTs for VO_2peak_ showed that no effects of pre-post intervention on the subgroups of middle-aged (mean difference 2.75, 95% CI −2.70 to 8.21, I^2^ 0%, 2 studies) and older-aged adults (mean difference 0.60, 95% CI −5.08 to 6.28, 1 study) were observed (Supplementary Figure S1).

Regarding subgroup analyses of RCTs for 6MWD, the results showed that the subgroup of patients undergoing aerobic exercise combined with resistance and respiratory exercise had a significantly greater 6MWD than those in the control program (SMD 0.91, 95% CI 0.53 to 1.29, one study), while there was a similar effect on 6MWD between the subgroup of patients undergoing aerobic plus resistance exercise and those undergoing a control program (SMD 0.82, 95% CI −0.72 to 2.37, I^2^ 0%, two studies) (Supplementary Figure S2a).

In subgroup analyses of non-RCTs for 6MWD, pooled effect estimates showed significant improvements in 6MWD among subgroups of young (mean difference 75.0, 95% CI 57.09 to 92.91, one study) and middle-aged adults (mean difference 99.75, 95% CI 25.20 to 174.30, I^2^ 0%, three studies), but not in the older-aged population (mean difference 75.0, 95% CI 57.09 to 92.91, one study) (Supplementary Figure S2b). In the subgroup analysis by exercise type, the 6MWD was significantly improved in subgroups performing aerobic exercise plus respiratory exercise (mean difference 75.0, 95% CI 57.09 to 92.91, one study) and aerobic exercise combined with resistance and respiratory exercise (mean difference 84.63, 95% CI 13.03 to 156.23, I^2^ 0%, four studies), but not in the subgroup performing aerobic plus resistance exercise (mean difference 144.4, 95% CI −7.62 to 296.42, one study) (Supplementary Figure S2c). For the subgroup analysis by exercise setting, pooled effect estimates illustrated that the 6MWD was significantly longer in a subgroup who underwent centre-based training (mean difference 76.29, 95% CI 58.75 to 93.82, I^2^ 0%, four studies), but not in those who performed telerehabilitation (mean difference 78.0, 95% CI −63.44 to 219.44, one study) and home-based training (mean difference 85.4, 95% CI −52.12 to 222.92, one study), when compared to the baseline (Supplementary Figure S2d).

In the sensitivity analyses, we removed studies affecting the influence plot. For the sensitivity analysis of non-RCTs for VO_2peak_, after removing the study by Ostrowska et al., 2023, the effect estimate was unchanged when compared to the main analysis (Supplementary Figure S3). For the sensitivity analysis of RCTs (after omitting the study by Amaral et al., 2022 [21]) and non-RCTs (after removing the study by Ahmed et al., 2021 [20]), for 6MWDs, the effect estimates were not changed when compared to the main analyses (Supplementary Figure S4a,b).

### 3.4. Publication Bias Assessment

The publication bias of the included studies of RCTs and non-RCTs for VO_2peak_ and 6MWD was assessed. No evidence of publication bias, based on a funnel plot, contour-enhanced funnel plot, and Begg’s and Egger’s tests of RCTs for VO_2peak_ and 6MWD, was found (Supplementary Figures S5 and S7). A possible publication bias of non-RCTs for VO_2peak_ and 6MWD was demonstrated by the asymmetry of a funnel plot falling in a low statistically significant area (*p* > 5%), as indicated by the contour-enhanced funnel plot. However, further investigation revealed no publication bias as there was no significance in Begg’s and Egger’s tests (Supplementary Figures S6 and S8).

### 3.5. Risk of Bias Assessment and Certainty of Evidence

RoB2 showed that most included RCTs had a high risk of bias [21,29,39], while the other two RCTs had a low risk of bias [40,41] (Supplementary Table S3). There were two domains contributing to the verdict of a high risk for each study: bias due to deviations from the intended intervention and bias in measurements of the outcome. For the domain of bias due to deviations from the intended intervention, using a per protocol was identified as high risk of bias. Regarding the bias in the measurement of the outcome, an unblinded outcome assessor was a factor introducing bias. For included non-RCTs, it was found by ROBINS-I that most included studies had a moderate risk of bias [20,23,42,43,44,45], while one study had a low risk of bias [22]. The main domain contributing to bias was the measurement outcome, which described the outcome assessor as being aware of the intervention given (Supplementary Table S4).

The certainty of evidence was evaluated to identify the degree of confidence that the effects estimate was correct. Our findings demonstrated that a cardiopulmonary exercise rehabilitation program may not be superior to the control program in improving VO_2peak_ and the 6MWD may not be improved after completing cardiopulmonary exercise rehabilitation. A cardiopulmonary exercise rehabilitation program may lead to a greater 6MWD than the control program, but the evidence for this is very uncertain. A cardiopulmonary exercise rehabilitation program may result in a large enhancement in the 6MWD after completion (Table 2).

## 4. Discussion

In this study, we aimed to determine whether exercise rehabilitation improves the CRF of long-COVID-19 survivors. The main findings of the meta-analysis provide scientific evidence that exercise rehabilitation (i) can improve the 6MWD and (ii) is superior in improving the 6MWD to control programs in long-COVID-19 survivors. This meta-analysis further highlighted more favourable effects in specific subgroups of (i) patients performing aerobic exercise combined with resistance and respiratory exercise, (ii) young and middle-aged adults, and (iii) patients undertaking a centre-based training approach for improving 6MWDs.

A reduction in CRF as a consequence of multi-organ impairments in long-COVID-19 survivors is recognised as a health condition linked to an increased cardiovascular risk. The findings of the current meta-analysis underscore the effectiveness of exercise rehabilitation on improving CRF, as reflected by the 6MWD of 76.46 m after completing the exercise program, and superior to the control program on CRF improvement, as reflected by the large effect size (0.85 SMD) of 6MWD in long-COVID-19 survivors. These findings are in agreement with previous systematic reviews and meta-analyses showing the beneficial effect of exercise rehabilitation on exercise capacity in a mixed population of acute to long-COVID-19 patients [28,46]. Other previous systematic reviews and meta-analyses revealed exercise rehabilitation’s positive effects on several health aspects, such as anxiety, depression, dyspnoea, and physical function [24,25,26,27]. Thus, our findings complement previous findings in terms of the beneficial effect of exercise rehabilitation on cardiorespiratory fitness and a particular population of long-COVID-19 patients.

A possible mechanism underlying the exercise training-induced improvement in cardiorespiratory fitness may be due to the coexistence of central and peripheral physiological adaptations (e.g., an improved chronotropic incompetence, an increased endothelial function, and arterial compliance) [47,48,49]. Our findings highlight that not only are there statistically significant differences in cardiorespiratory fitness, but also clinically meaningful changes, as the 6MWD exceeds the minimal clinically important difference (MCID) of 30 m reported in patients with chronic respiratory disease [50]. However, this should be interpreted with caution since the MCID of 30 m was determined from the patient group other than long-COVID-19 survivors. Further studies should be performed regarding this point.

The health benefits of exercise rehabilitation depend on exercise-component-specific responses. Subgroup analyses in our study appeared to have statistically significant differences in the 6MWD in the subgroups of patients undergoing (i) aerobic exercise plus resistance and respiratory exercise and (ii) a centre-based training approach. Our results showed that aerobic exercise combined with resistance and respiratory exercise had a positive effect on the 6MWD (an improved distance of 84.6 m) and had a larger positive effect on the 6MWD when compared to the control program (0.91 SMD). The 6MWD represents a functional capacity that requires multiple organ systems (i.e., cardiovascular, pulmonary, and skeletal systems) to reflect its ability [51]. Long-COVID-19 survivors not only experience an impaired pulmonary function, but the cardiovascular and even peripheral muscular systems are also impaired [7,8]. Aerobic and respiratory exercises, reflecting a cardiopulmonary exercise regimen, have been suggested to improve the cardiopulmonary capacity in patients with chronic respiratory disease [17]. The greater improvement in the 6MWD among long-COVID-19 survivors in the group who performed additional resistance exercises may be attributed to the comprehensive enhancement in cardiopulmonary and peripheral muscular functions. Regarding exercise approaches, our meta-analysis identified a better improvement in 6MWDs in a subgroup of patients conducting centre-based exercise. Exercise under supervision is relatively easier to follow and adhere to rather than unsupervised approaches [52]. Thus, our finding suggests that supervised exercise in a centre-based setting is suitable for improving cardiorespiratory fitness in long-COVID-19 survivors.

In addition to exercise-component-specific responses, our subgroup analysis showed that age was a factor affecting cardiorespiratory fitness responses after completing exercise rehabilitation. The subgroup findings appeared to indicate a favourable effect of exercise rehabilitation on 6MWDs in the young to middle-aged adults, but not in older-aged adults. The lack of statistical difference in 6MWDs between groups in the older-aged adult subgroup is likely explained by a low adherence rate to exercise training. Existing evidence suggests that older-aged adults often experience severe post-COVID-19 symptoms [53]. It is possible that the low exercise adherence rate is attributed to these severe long-COVID-19 symptoms in older adults, resulting in insufficient power to induce physiological changes in cardiorespiratory fitness [41]. One included study investigated delivering exercise rehabilitation in a home-based approach in older adults [45]. This may be an additional factor contributing to the low exercise adherence rate; however, the authors did not report the exercise adherence rate. Additionally, low exercise adherence rate may be attributable to some comorbidities (e.g., hypertension) in older-aged adults [54]. In this review, hypertension rates were 56% and 36% in subgroups of older-aged adults and young to middle-aged adults, respectively. Higher rates of comorbidities in older-aged adults may promote a reduction in exercise tolerance [54], thereby leading to low exercise adherence.

Compared to the baseline 6MWD value, low certainty of evidence suggests that exercise rehabilitation may result in a large increase in 6MWD. The certainty of evidence was downgraded two levels due to a serious risk of bias and imprecision. For the serious risk of bias, an unblinded assessor measuring the outcome was suspected to be a factor lowering the reliability of the finding. A wide range of confidence intervals, reflecting this imprecision, additionally promoted the low certainty.

Despite the significantly longer 6MWD in exercise rehabilitation, it is uncertain whether the exercise rehabilitation program has a superior effect on cardiorespiratory fitness (as measured by 6MWD) in this patient group when compared to the control program due to the very low certainty of evidence. Two factors contributing to a reduction in evidence certainty were the moderate risk of bias and imprecision. For the moderate risk of bias, participant’s awareness of the treatment given, inappropriate analysis (using a per protocol) [55], and unblinded outcome measurements were identified as problematic, leading to deviations in findings. Furthermore, a very wide range of confidence intervals is also considered to imprecision. Given these considerations, the findings should be interpreted with caution.

The strengths of the current meta-analysis are as follows: We attempted to prevent potential biases through an exhaustive search of available evidence (not restricted by language), through using rigorous review processes conducted by independent reviewers, and through careful consideration of evidence quality. Some limitations, however, should be acknowledged. First, considerably heterogeneity in the 6MWD (I^2^ 76.4%) was detected due to clinical heterogeneity (i.e., different participant characteristics and different exercise regimens). Such heterogeneity was determined to be modest after carrying out a subgroup analysis. However, the subgroup analyses limit our ability to draw a strong conclusion on whether exercise rehabilitation is superior in improving cardiorespiratory fitness compared to the control program. Second, some potential biases resulting from an uncontrolled process within individual publications are observed; more than 50% of RCTs and 80% of non-RCTs had a high risk of bias and moderate risk of bias, respectively. Third, the sample size in most studies was small. Consequently, the restricted sample size impedes statistical power detection. Lastly, the number of relevant studies is low. There were particularly a limited number of studies investigating the effect of exercise rehabilitation on VO_2peak_ (two RCTs and three non-RCTs). The insufficient number of studies, together with the inadequate sample size, could limit the statistical power to detect differences in VO_2peak_ between patients in the exercise rehabilitation and control program groups. Thus, more studies with a comprehensive research design (especially RCTs) and large sample sizes are warranted.

## 5. Conclusions

The meta-analysis findings highlight that exercise rehabilitation is effective for improving cardiorespiratory fitness, as measured by the 6MWD (but not the VO_2peak_) in long-COVID-19 survivors. Specifically, in young to middle-aged adults, aerobic exercises combined with resistance and respiratory exercise and centre-based approaches are likely to yield greater benefits with regard to cardiorespiratory fitness.

## 6. Clinical Implication

Considering the low to very low certainty of evidence, our confidence in supporting the use of exercise rehabilitation to improve the cardiorespiratory fitness in long-COVID-19 survivors is reduced. Consequently, we are limited in our ability to recommend or not recommend interventions in clinical practice.

## Figures and Tables

**Table 1:** Characteristics of included studies.

Author (Year)	Design	Participants	COVID-19 Characteristics	Region	IG Program	CG Program	Mean-Aged Population	Outcome and Results
Ahmed (2022) [20]	non-rct	IG: *n* = 20 (65% M) age = 39.6 ± 2.4 y Comorbidities (N/A) No control group	Hospitalisation duration IG: 16 days No control group Long-COVID-19 duration IG: 5 wk No control group	Asia	Aerobic: 50–70% of HR_max_ or RPE 4–6 of 10 scales Resistance: - Respiratory: breathing Others: - (20–60 min, 3 days, 5 wk) Centre-based training	-	Young-aged adults	↑ 6MWD after completing training
Amaral (2022) [21]	rct	IG: *n* = 12 (58% M)20 age = 51.9 ± 10.2 y Comorbidities (42% HT, 33% DM, 8%DLP) CG: *n* = (40% M) age = 53.3 ± 11.6 y Comorbidities (55% HT, 5% DM, 10%DLP)	Hospitalisation duration IG: 6 days CG: 7 days Long-COVID-19 duration IG: 5.1 wk CG: 5.1 wk	South America	Aerobic: 11–13RPE Resistance: intensity not reported Respiratory: - Others: - (30 min of aerobic exercise, 2–5 days, 12 wk) Telerehabilitation	No intervention	Middle-aged adults	↔ 6MWD compared to CG
Calvo-Paniagua (2022) [22]	non-rct	IG: *n* = 68 (38% M) age = 48.5 ± 9.7 y Comorbidities (1.5% HT, 8.8% DM) No control group	Hospitalisation duration IG: 7.7 days No control group Long-COVID-19 duration IG: 12 wk No control group	Europe	Aerobic: no intensity Resistance: intensity not reported Respiratory: intensity not reported Others: - (40 min, 3 days, 7 wk) Telerehabilitation	-	Middle-aged adults	↑ 6MWD after completing training
Compagno (2022) [23]	non-rct	IG: *n* = 30 (65% M) age = 58.4 ± 11.6 y Comorbidities (33.3% HT, 10% DM, 30%DLP) No control group	Hospitalisation duration IG: N/A No control group Long-COVID-19 duration IG: 12 wk No control group	Europe	Aerobic: 60–80% VO_2peak_ Resistance: 30–50% of the 1-RM Respiratory: - Others: - (90 min, 3 days, 4 wk) Centre-based training	-	Middle-aged adults	↑ VO_2peak_ after completing training
Everaerts (2020) [42]	non-rct	IG: *n* = 22 (68% M) age = 54.2 ± 10.4 y Comorbidities (N/A) No control group	Hospitalisation duration IG: 26.7 days No control group Long-COVID-19 duration IG: 10 wk No control group	Europe	Aerobic: 60–75% individuals exhibited maximum capacity Resistance: intensity not reported Respiratory: - Others: - (90 min, 3 days, 12 wk) Centre-based	-	Middle-aged adults	↑ 6MWD and VO_2peak_ after completing training
Furtado (2023) [39]	rct	IG: *n* = 16 (50% M) age = 47.5 ± 12 y Comorbidities (43.8% HT, 12.5% DM, 37.5%DLP) CG: *n* = 16 (37.5% M) age = 49.2 ± 13 y Comorbidities (12.5% HT, 18.8% DM, 31.2%DLP)	Hospitalisation duration IG: N/A CG: N/A Long-COVID-19 duration IG: 5 wk CG: 5 wk	South America	Aerobic: 6–8 Borg scale of 10 scales Resistance: 6–8 Borg scale of 10 scales Respiratory: - Others: neuromuscular exercise (45–60 min, 3 days, 8 wk) Telerehabilitation	No physical training program	Middle-aged adults	↑ 6MWD compared to CG
Gloeckl (2021) [43]	non-rct	IG: *n* = 50 (44% M) age = 58.7 ± 7.5 y Comorbidities (42%HT, 14%DM, 26%DLP) No control group	Hospitalisation duration IG: 38.3 days No control group Long-COVID-19 duration IG: 8.5 wk No control group	Asia	*Aerobic:* 60–70% of peak power *Resistance:* intensity not reported *Respiratory:* breathing *Others:* - (20 min for aerobic exercise, 30 min for resistance exercise, 30 min for respiratory exercise, 5 days, 3 wk) Centre-based training	-	Middle-aged adults	↑ 6MWD after completing training
Jimeno-Almazán (2023) [29]	rct	IG: *n* = 60 (68.3% M) age = 43.8 ± 8.1 y Comorbidities (N/A) CG: *n* = 20 (70% M) age = 47.8 ± 7.6 y Comorbidities (N/A)	Hospitalisation duration IG: N/A CG: N/A Long-COVID-19 duration IG: 44.3 wk CG: 39.4	Europe	*Aerobic:* 70–80%HRR or 16 Borg scale, 3–5 min/55–65%HRR, 2–3 min *Resistance:* 50%RM *Respiratory:* inspiratory muscle training *Others:* - (Varied session: 3 days of aerobic resistance and inspiratory muscle training every day, 8 wk) Centre-based training	Self-management	Middle-aged adults	↔ VO_2peak_ compared to CG
Li (2021) [40]	rct	IG: *n* = 59 (45.8% M) age = 49.2 ± 10.8 y Comorbidities (13.6% HT, 13.6% DM) CG: *n* = 60 (43.3% M) age = 52.0 ± 11.1 y Comorbidities (30% HT, 15% DM)	Hospitalisation duration IG: 28.7 days CG: 23.7 days Long-COVID-19 duration IG: 11 wk CG: 11.2 wk	Asia	*Aerobic:* 30–40% HRR to 40–60% HRR *Resistance:* intensity not reported *Respiratory:* breathing exercise *Others:* - (40–60 min, 3–4 days, 6 wk) Telerehabilitation	Education	Middle-aged adults	↑ 6MWD compared to CG
Longobardi (2023) [41]	rct	IG: *n* = 25 (48% M) age = 60.8 ± 7.1 y Comorbidities (60% HT, 32% DM, 52%DLP) CG: *n* = 25 (52% M) age = 61.2 ± 7.7 y Comorbidities (52% HT, 40% DM, 56%DLP)	Hospitalisation duration IG: 18 days CG: 19 days Long-COVID-19 duration IG: 25.4 wk CG: 25.1 wk	South America	*Aerobic:* Borg scale (9–11) and Borg scale (15–17) *Resistance:* Borg scale (9–11) and Borg scale (15–17) *Respiratory:* - *Others:* - (60–80 min, 3 days, 16 wk) Home-based training	General active lifestyle	Older-aged adults	↔ VO_2peak_ compared to CG
Ostrowska (2023) [44]	non-rct	IG: *n* = 97 (45% M) age = 59.3 ± 13.3 y Comorbidities (46.4% HT, 27.7%DLP) No control group	Hospitalisation duration IG: N/A Long-COVID-19 duration IG: 12 wk	Europe	*Aerobic:* intensity not reported *Resistance:* intensity not reported *Respiratory:* - *Others:* - (90 min, 3 days, 6 wk) Centre-based training	-	Older-aged adults	↑ 6MWD after completing training ↔ VO_2peak_ after completing training
Stavrou (2021) [45]	non-rct	IG: *n* = 20 (75% M) age = 64.1 ± 9.9 y Comorbidities (65% HT, 20%DM) No control group	Hospitalisation duration IG: 15.1 days Long-COVID-19 duration IG: 8 wk	Europe	*Aerobic:* 75–110%HR_peak_ *Resistance:* intensity not reported *Respiratory:* Yoga breathing *Others:* - (100 min, 3 days, 8 wk) Home-based training	-	Older-aged adults	↑ 6MWD after completing training

Age is presented as mean ± standard deviation. CG, control group; DLP, dyslipidaemia; DM, diabetes; HR_max_, maximum heart rate; HRR, heart rate reserve; HT, hypertension; IG, intervention group; M, male; min, minute; N/A, not applicable; *n*, number; non-rct, non-randomised controlled trial; rct, randomised controlled trial; RPE, rating perceived exertion; VO_2peak_, peak oxygen consumption; wk, week; 1-RM, one repetition maximum; 6MWD, six-minute walk distance; y, year; ↑, represents an improvement in outcomes; ↔, represents a comparable result.

**Table 2:** Summary of findings.

Outcomes	Mean Change (SD)		SMD (95% CI)	Number of Participants (Studies)	Certainty of Evidence (GRADE)	Comments
	Control Program	Exercise Rehabilitation Program				
Peak oxygen consumption (VO_2peak_, mL/min/kg)	Mean change VO_2peak_ was 1.75 (5.4)	Mean change VO_2peak_ was 0.35 (6.2)	0.26 (−0.11 to 0.64)	130 (two RCTs)	ΘΘΘΘ Very low ^a,b^	Exercise rehabilitation program may not be superior in improving VO_2peak_ compared to control program
Six-minute-walk distance (6MWD, m)	Mean change 6MWD was 14.4 (62.6)	Mean change 6MWD was 74.4 (75.6)	0.85 (0.11 to 1.59)	183 (three RCTs)	ΘΘΘΘ Very low ^a,b^	Exercise rehabilitation program may provide a greater 6MWD than the control program, but the evidence is very uncertain
**Outcomes**	**Mean (SD)**		**MD (95% CI)**	**Number of Participants (studies)**	**Certainty of Evidence (GRADE)**	**Comments**
	**Pre-Intervention**	**Post-Intervention**				
Peak oxygen consumption (VO_2peak_, mL/min/kg)	Mean VO_2peak_ was 17 (4.3)	Mean VO_2peak_ was 19.9 (6.0)	1.72 (−2.21 to 5.66)	92 (three non-RCTs)	⊕ΘΘΘ Very low ^c,d^	Exercise rehabilitation program may not improve VO_2peak_
Six-minute walk distance (6MWD, m)	Mean 6MWD was 455.8 (156.8)	Mean 6MWD was 543.9 (174.3)	76.45 (59.19 to 93.71)	277 (six non-RCTs)	⊕⊕ΘΘ Low ^c,e^	Exercise rehabilitation program may result in a large increase in 6MWD

CI, confidence interval; GRADE, Grading of Recommendations, Assessment, Development and Evaluation; m, meter; MD, mean difference; mL/min/kg, milliliter per kilogram per minute; non-RCTs, non-randomised controlled trials; RCTs, randomised controlled trials; SD, standard deviation; SMD, standardised mean difference. ^a^ Downgraded one level due to moderate risk of bias. ^b^ Downgraded three levels due to imprecision (95% CI crossed three thresholds; trivial to moderate effect). ^c^ Downgraded one level due to serious risk of bias. ^d^ Downgraded two levels due to imprecision (95% CI crossed two thresholds; small to moderate effects). ^e^ Downgraded one level due to imprecision (95% CI crossed one threshold; large effect). ⊕⊕⊕⊕, High certainty; ⊕⊕⊕Θ, Moderate certainty; ⊕⊕ΘΘ, Low certainty; ⊕ΘΘΘ, Very low certainty; ΘΘΘΘ, Very low certainty.

## Data Availability

All relevant data are within the paper and its Supplementary Materials.

## Supplementary Materials

The following supporting information can be downloaded at: https://www.mdpi.com/article/10.3390/jcm13123621/s1, Table S1: Search strategy; Table S2: Excluded studies; Table S3: A revised Cochrane risk-of-bias tool for randomised trials (RoB 2); Table S4: Risk of bias in non-randomised studies-of interventions (ROBINS-I); Figure S1: Subgroup analysis of non-randomised controlled trials by mean-aged population. CI, confidence interval; Effect, weighted mean difference; VO_2peak_, peak oxygen consumption; Figure S2: (a) Subgroup analysis of randomised controlled trials by exercise type. CI, confidence interval; N, number of participants; SD, standard deviation; 6MWD, six-minute walk distance; SMD, standardised mean difference; 1, aerobic plus resistance exercise; 2, aerobic combined with resistance and respiratory exercise; (b) Subgroup analysis of non-randomized controlled trials by mean-aged population. CI, confidence interval; Effect, weighted mean difference; 6MWD, six-minute walk distance; (c) Subgroup analysis of non-randomised controlled trials by exercise type. CI, confidence interval; Effect, weighted mean difference; 6MWD, six-minute walk distance; 1, aerobic plus resistance exercises; 2, aerobic plus respiratory exercises; 3, aerobic combined with resistance and respiratory exercises; (d) Subgroup analysis of non-randomised controlled trials by exercise setting. CI, confidence interval; Effect, weighted mean difference; 6MWD, six-minute walk distance; Figure S3: Sensitivity analysis of non-randomised controlled trials for VO_2peak_ after omitting Ostrowska et al., 2023. CI, confidence interval; Effect, weighted mean difference; VO_2peak_, peak maximum consumption; Figure S4: (a) Sensitivity analysis of randomised controlled trials for 6MWD after omitting Amaral et al., 2022 [21]. CI, confidence interval; N, number of participants; SD, standard deviation; SMD, standardised mean difference; 6MWD, six-minute walk distance; (**b**) Sensitivity analysis of non-randomised controlled trials for 6MWD after omitting Ahmed et al., 2021 [20]. CI, confidence interval; Effect, weighted mean difference; 6MWD, six-minute walk distance; Figure S5: Publication bias of randomised controlled trials for VO_2peak_. (a) Funnel plot and Begg’s test; (b) Contour-enhanced funnel plot, CI, confidence interval; se, standard error; SMD, standard mean difference; VO_2peak,_ peak oxygen consumption; Figure S6: Publication bias of non-randomised controlled trials for VO_2peak_. (a) Funnel plot; (b) Contour-enhanced funnel plot; (c) Egger’s graph and Begg’s test, CI, confidence interval; se, standard error; SND, standard normal deviate; WMD, weighted mean difference; VO_2peak_, peak oxygen consumption; Figure S7: Publication bias of randomised controlled trials for 6MWD. (a) Funnel plot; (b) Contour-enhanced funnel plot; (c) Egger’s graph and Begg’s test, CI, confidence interval; se, standard error; SMD, standardised mean difference; SND, standard normal deviate; 6MWD, six-minute walk distance; Figure S8: Publication bias of randomised controlled trials for 6MWD. (a) Funnel plot; (b) Contour-enhanced funnel plot; (c) Egger’s graph and Begg’s test, CI, confidence interval; se, standard error; SMD, standardised mean difference; SND, standard normal deviate; 6MWD, six-minute walk distance. References [56,57,58,59,60,61,62,63,64,65,66,67,68,69,70,71,72,73,74,75,76,77,78,79,80,81,82,83,84,85,86,87,88,89,90,91,92,93,94,95,96,97,98,99,100] are cited in supplementary materials.
