# Supplementary material for: Effects of Exercise Rehabilitation on Cardiorespiratory Fitness in Long-COVID-19 Survivors: A Meta-Analysis
Source: J Clin Med. 2024 Jun 20;13(12):3621. doi: 10.3390/jcm13123621 (PMC11204731; doi:10.3390/jcm13123621)
Supplement: Supplementary file 1 [file jcm-13-03621-s001.zip › jcm-3000068-supplementary.pdf]

Table S1 Search strategy

Databases	Steps	Search terms	Results
**PubMed**	#1	('covid'[Title/Abstract] OR 'coronavirus'[Title/Abstract])	385,336
	#2	('cardiorespiratory fitness'[Title/Abstract] OR 'physical fitness'[Title/Abstract] OR 'cardiopulmonary fitness'[Title/Abstract] OR 'fitness performance'[Title/Abstract] OR 'cardiorespiratory performance'[Title/Abstract])	47,696
	#3	#1 AND #2 Sort by: Best Match	**509**
**CINAHL**	#S1	AB ‘covid’ OR AB ‘coronavirus’	61,966
	#S2	AB cardiorespiratory fitness OR AB physical fitness OR AB cardiopulmonary fitness OR fitness performance OR AB cardiorespiratory performance	13,227
	#S3	(AB #S1) AND (AB #S2)	**122**
**Scopus**	#1	TITLE-ABS-KEY (‘covid’ OR ‘coronavirus’)	577,065
	#2	TITLE-ABS-KEY ('cardiorespiratory fitness' OR 'physical fitness' OR 'cardiopulmonary fitness' OR 'fitness performance' OR 'cardiorespiratory performance')	52,182
	#3	#1 AND #2	**652**
**Embase**	#1	‘covid’/exp ‘covid’ OR ‘coronavirus’:ab,ti	397,868
	#2	'cardiorespiratory fitness' OR 'physical fitness' OR 'cardiopulmonary fitness' OR 'fitness performance' OR 'cardiorespiratory performance':ab,ti	34,632
	#3	#1 AND #2	**463**

Databases	Steps	Search terms	Results
**Cochrane**	#1	(‘covid’):ti,ab,kw OR (‘coronavirus’): ti,ab,kw	15,530
	#2	('cardiorespiratory fitness'): ti,ab,kw OR ('physical fitness'):ti,ab,kw OR ('cardiopulmonary fitness'):ti,ab,kw OR ('fitness performance'):ti,ab,kw OR ('cardiorespiratory performance'):ti,ab,kw	10,329
	#3	#1 AND #2	**147**

Table S2 Excluded studies

References	Reason for exclusion
ACTRN12622000742774 (2022) [56]	Protocol
Ahmed (2020) [57]	Protocol
Anastasio (2022) [58]	Observational study
Aparisi (2021) [59]	Observational study
Arena (2021) [60]	Review
Azmodeh (2022) [61]	Observational study
Back (2022) [10]	Observational study
Baricich (2021) [62]	Observational study
Bellmunt (2021) [63]	Protocol
Blokland (2020) [64]	Observational study
Bohn (2021) [65]	Investigating in older adults without post- and long-COVID-19 infection
Botek (2022) [66]	No exercise intervention
Brandenburg (2021) [67]	Observational study
Brawner (2021) [68]	No CRF outcome
Cafiero (2021) [69]	Observational study
Cancino-López (2021) [70]	Observational study
Christensen (2021) [71]	Observational study

References	Reason for exclusion
Crameri (2020) [72]	Observational study
CTRI (2021) [73]	Protocol
Cuenca-Zaldivar (2022) [74]	No CRF outcome
Debeaumont (2021) [75]	Observational study
Delevatti (2022) [76]	Protocol
Drks (2022) [77]	Protocol
Fereydounnia (2022) [78]	No exercise intervention
Gattoni (2022) [79]	Observational study
Geijerstam (2021) [80]	Observational study
Ekblom-Bak (2021) [11]	Observational study
Hail University Poly Clinic (2022) [81]	Protocol
Hekmatikar (2021) [82]	Protocol
Hermann (2020) [83]	Investigating in participants without post- and long-COVID-19 infection
Heubel (2020) [84]	Protocol
Jafarnezhadgero (2022) [85]	Observational study
Jimeno-Almazán (2021) [86]	Observational study
Jimeno-Almazán (2022) [87]	Observational study
Kisiel (2022) [88]	Observational study
Ladlow (2022) [12]	Observational study

References	Reason for exclusion
Millet (2022) [89]	Observational study
Milovancev (2021) [90]	Observational study
Mitropoulos (2022) [91]	Observational study
Pleguezuelos (2023) [92]	Unavailable numerical data
Schaeffer (2022) [93]	Observational study
Skjørten (2021) [94]	Observational study
Stavrou (2022) [95]	Observational study
Trapé (2021) [96]	Protocol
Williamson (2021) [97]	No full text
Windt (2022) [98]	Unavailable numerical data
Wood (2022) [99]	Observational study
Xiong (2021) [100]	Observational study

Table S3 A revised Cochrane risk-of-bias tool for randomized trials (RoB 2)

Author (year)	RoB 2						
	Domain 1: Risk of bias arising from the randomization process	Domain 2: Risk of bias due to deviations from the intended interventions (*effect of assignment to intervention*)	Domain 2: Risk of bias due to deviations from the intended interventions (*effect of adhering to intervention*)	Domain 3: Missing outcome data	Domain 4: Risk of bias in measurement of the outcome	Domain 5: Risk of bias in selection of the reported result	Overall risk of bias
**Amaral (2022) [21]**	+	-	+	+	+	+	**-**
**Furtado (2023) [39]**	+	-	-	+	-	-	**-**
**Li (2021) [40]**	+	+	+	+	+	+	**+**
**Longobardi (2023) [41]**	+	+	+	+	+	+	**+**
**Jimeno-Almazán (2023) [29]**	+	?	+	+	-	+	**-**

Judgement: -, High; ?, Unclear; +, Low

Table S4 Risk of bias in non-randomized studies-of interventions (ROBINS-I)

Author (year)	ROBINS-I							
	Domain 1: Bias due to confounding	Domain 2: Bias in selection of participants into the study	Domain 3: Bias in classification of interventions	Domain 4: Bias due to deviations from intended interventions	Domain 5: Bias due to missing data	Domain 6: Bias in measurement of outcomes	Domain 7: Bias in selection of the reported result	Overall bias
**Ahmed (2022) [20]**	L	L	L	L	L	M	L	**M**
**Calvo-Paniagua (2022) [22]**	L	L	L	L	L	L	L	**L**
**Compagno (2022) [23]**	L	L	L	L	L	M	L	**M**
**Everaerts (2020) [42]**	L	L	L	L	L	M	L	**M**
**Gloeckl (2021) [43]**	L	L	L	L	L	M	L	**M**
**Ostrowska (2023) [44]**	L	L	L	NI	L	L	L	**M**
**Stavrou (2021) [45]**	L	L	L	L	L	M	L	**M**

Judgement: L, Low; M, Moderate; NI, No information

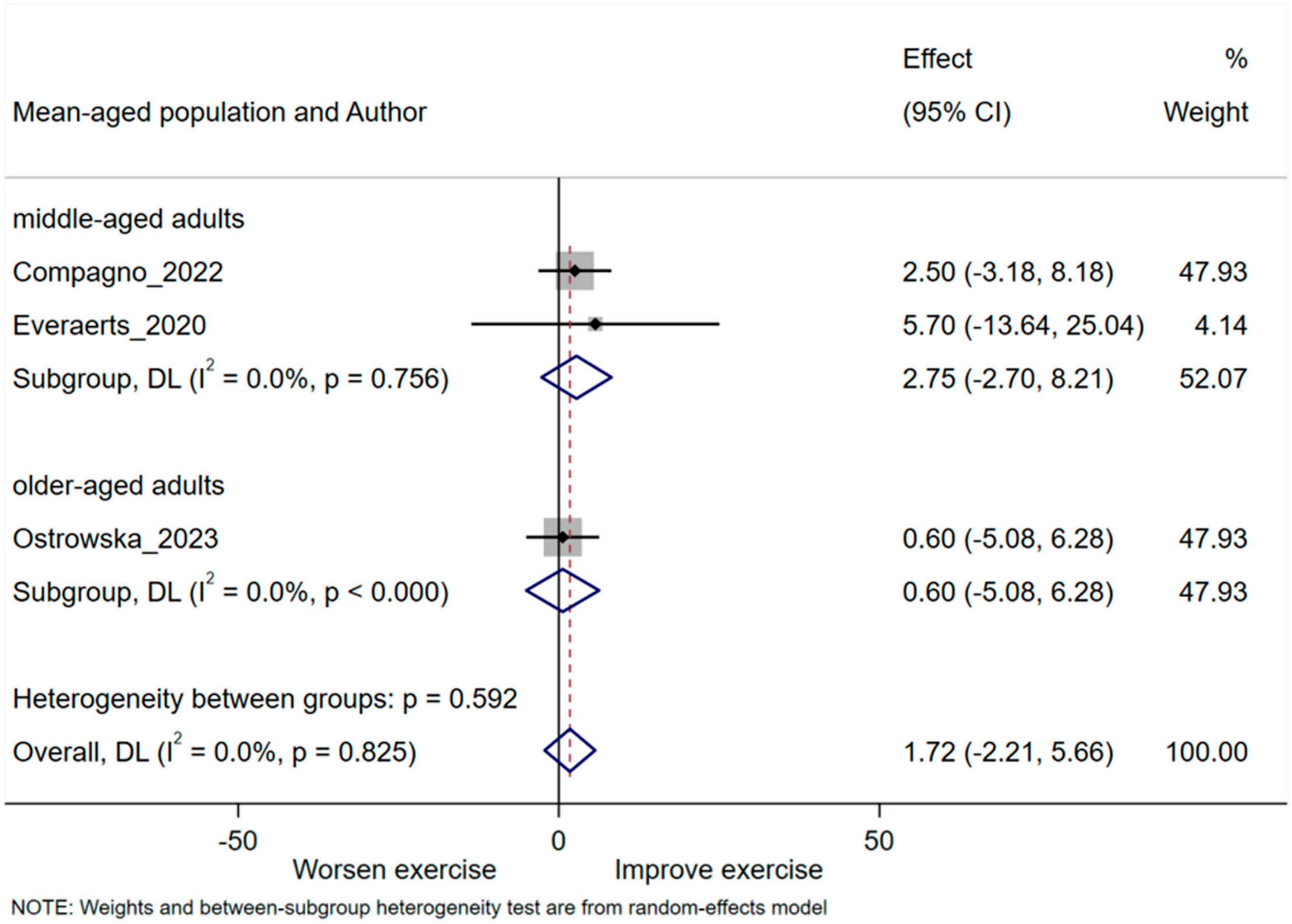

Figure S1 Subgroup analysis of non-randomized controlled trials by mean-aged population. CI, confidence interval; Effect, weighted mean difference; VO_{2peak}, peak oxygen consumption [23,42,44].

Figure S2 Subgroup analysis for 6MWD

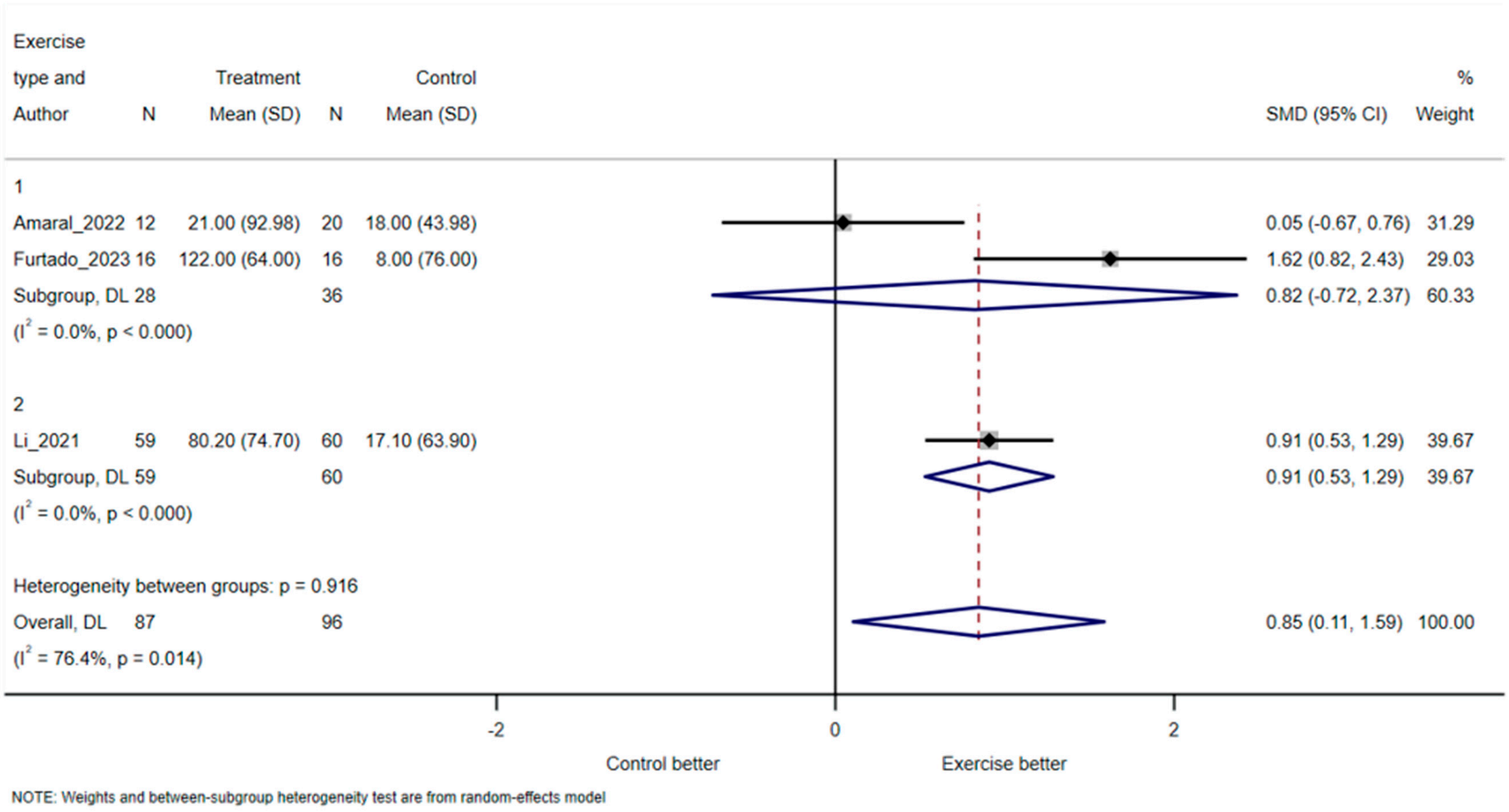

Figure S2a Subgroup analysis of randomized controlled trials by exercise type. CI, confidence interval; N, number of participants; SD, standard deviation; 6MWD, six-minute walk distance; SMD, standardized mean difference; 1, aerobic plus resistance exercise; 2, aerobic combined with resistance and respiratory exercise [21,39,40].

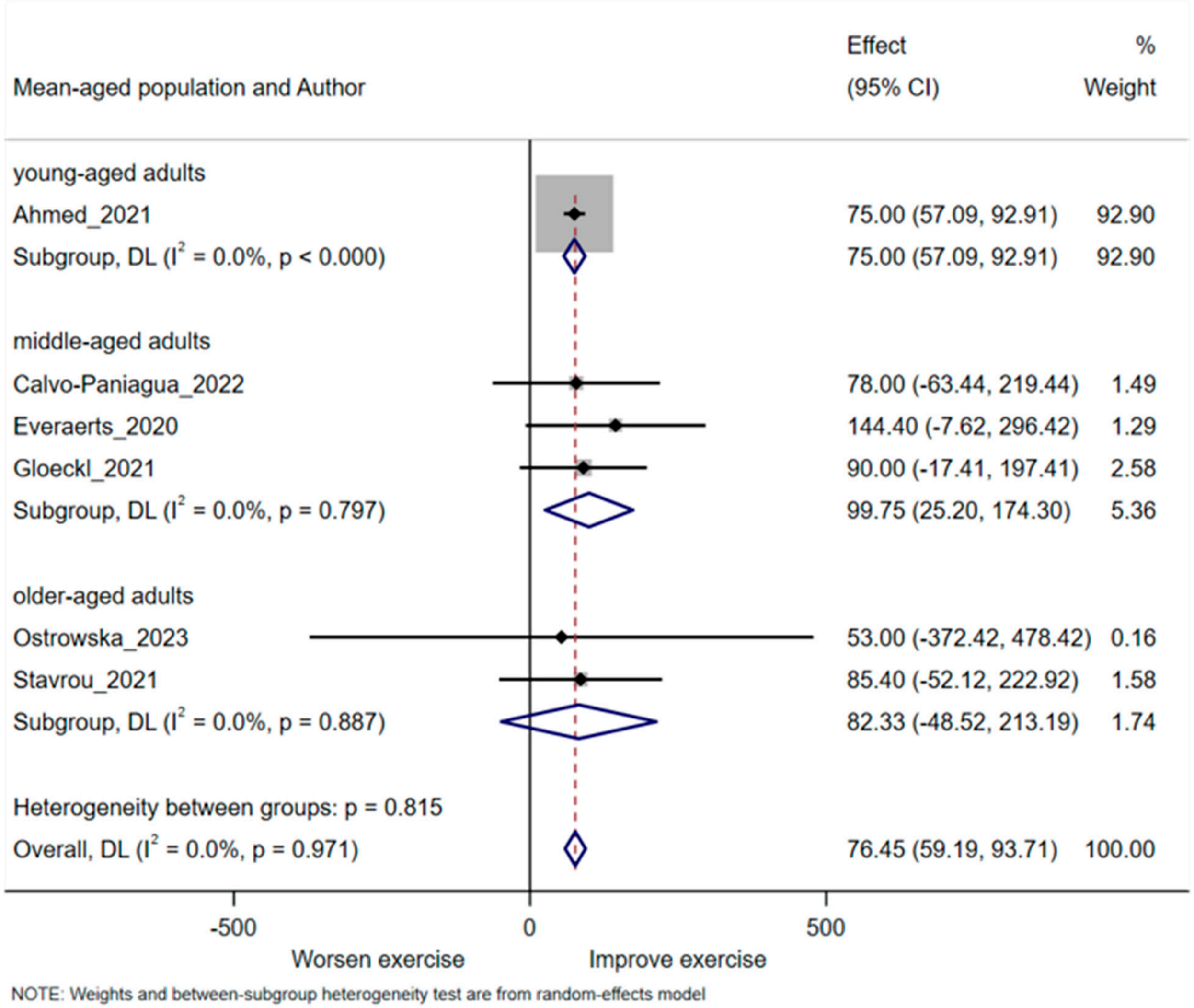

Figure S2b Subgroup analysis of non-randomized controlled trials by mean-aged population. CI, confidence interval; Effect, weighted mean difference; 6MWD, six-minute walk distance [20,22,42,43,44,45].

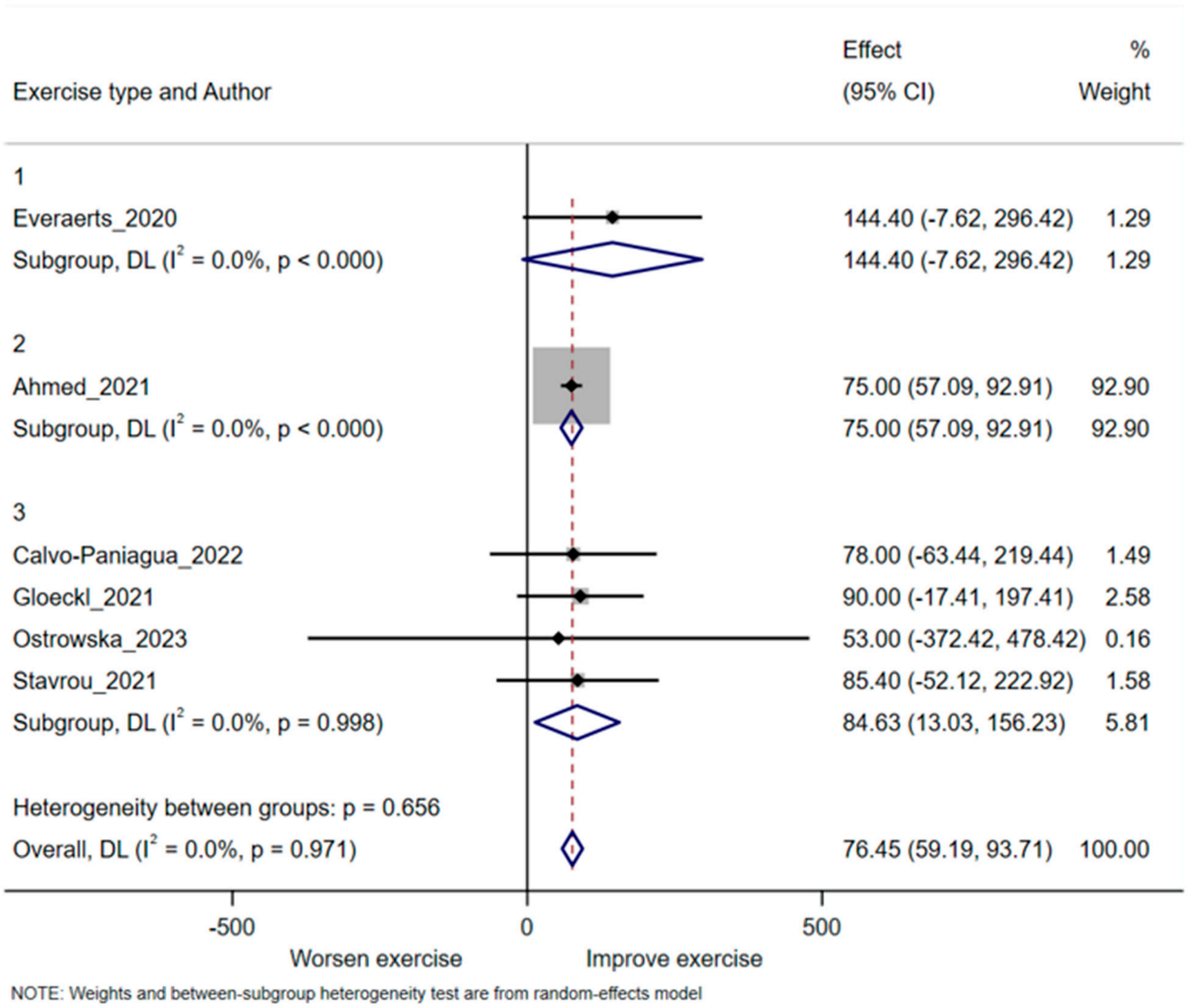

Figure S2c Subgroup analysis of non-randomized controlled trials by exercise type. CI, confidence interval; Effect, weighted mean difference; 6MWD, six-minute walk distance; 1, aerobic plus resistance exercises; 2, aerobic plus respiratory exercises; 3, aerobic combined with resistance and respiratory exercises [20,22,42,43,44,45].

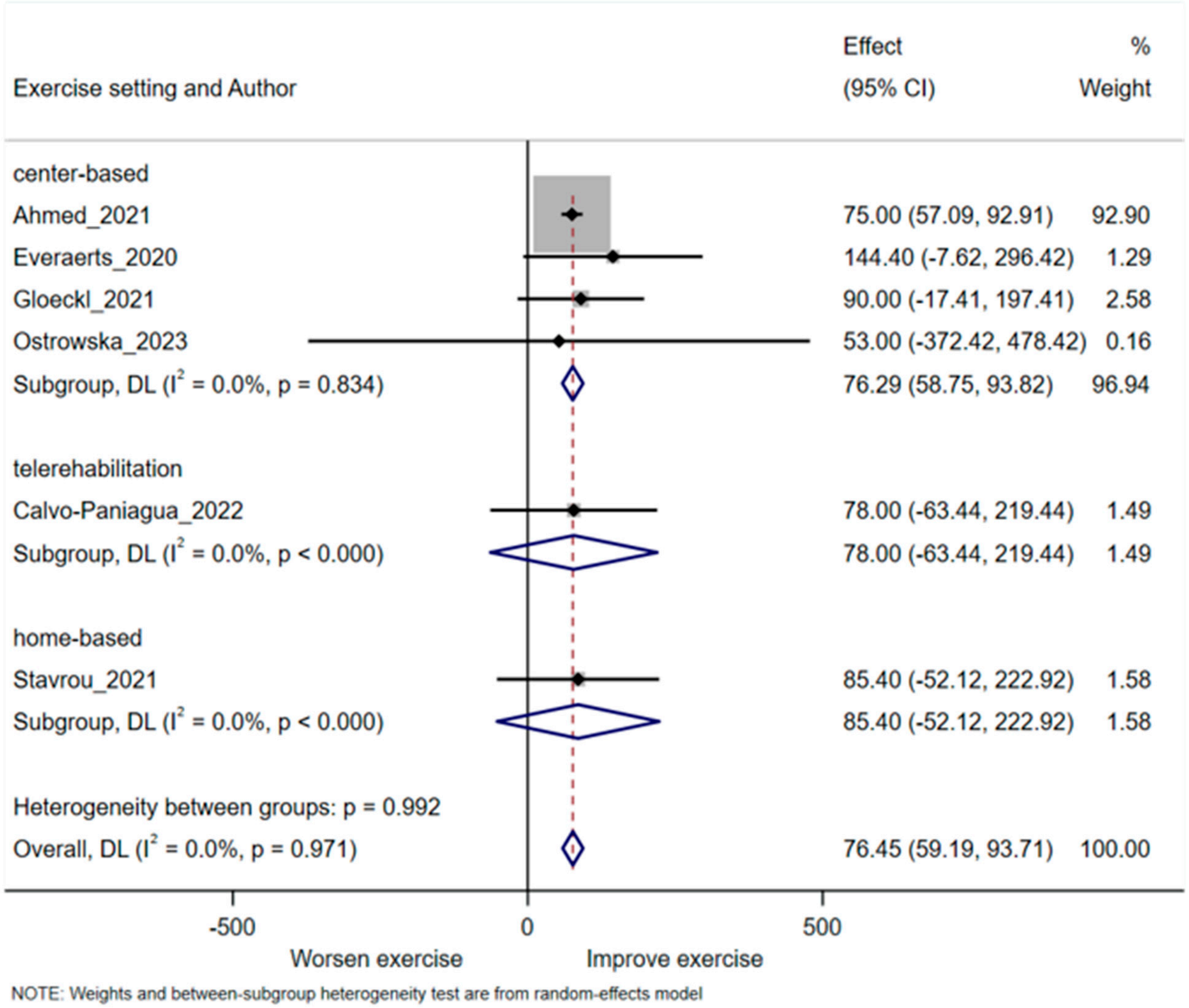

Figure S2d Subgroup analysis of non-randomized controlled trials by exercise setting. CI, confidence interval; Effect, weighted mean difference; 6MWD, six-minute walk distance [20,22,42,43,44,45].

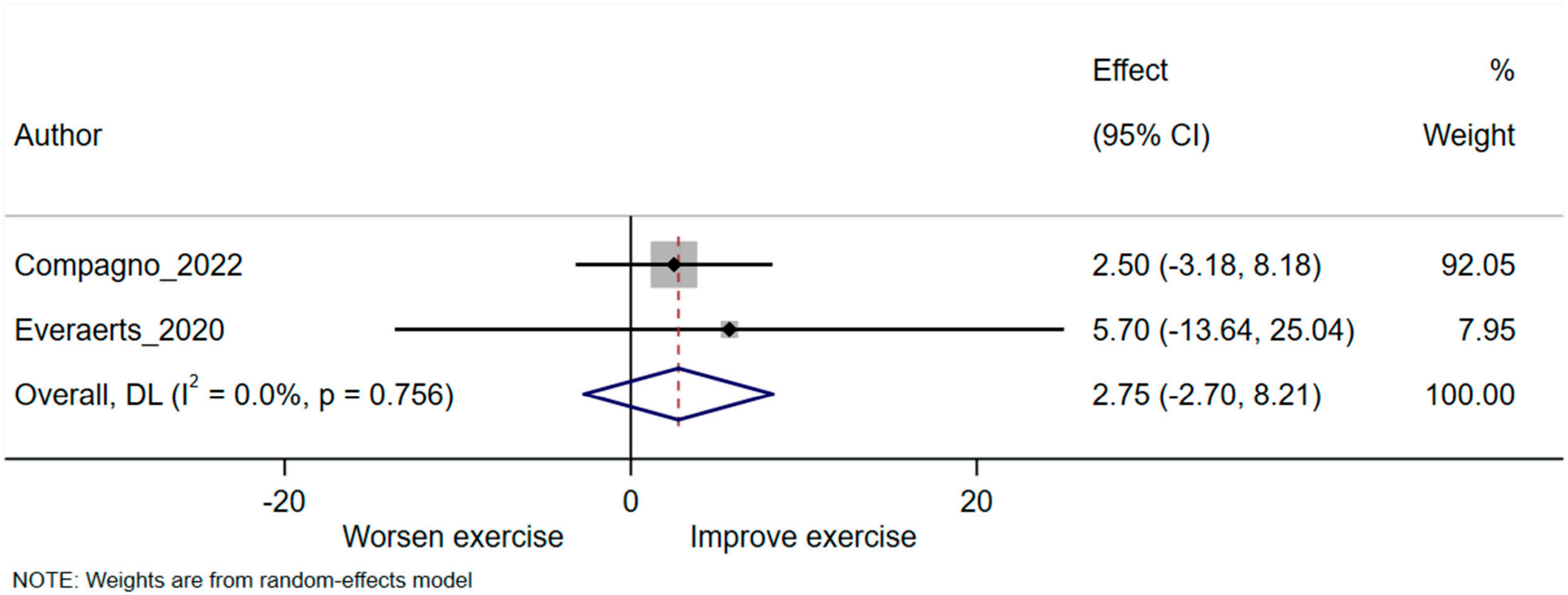

Figure S3 Sensitivity analysis of non-randomized controlled trials for VO_{2peak} after omitting Ostrowska et al., 2023. CI, confidence interval; Effect, weighted mean difference; VO_{2peak}, peak maximum consumption [23,42].

Figure S4 Sensitivity analysis for 6MWD

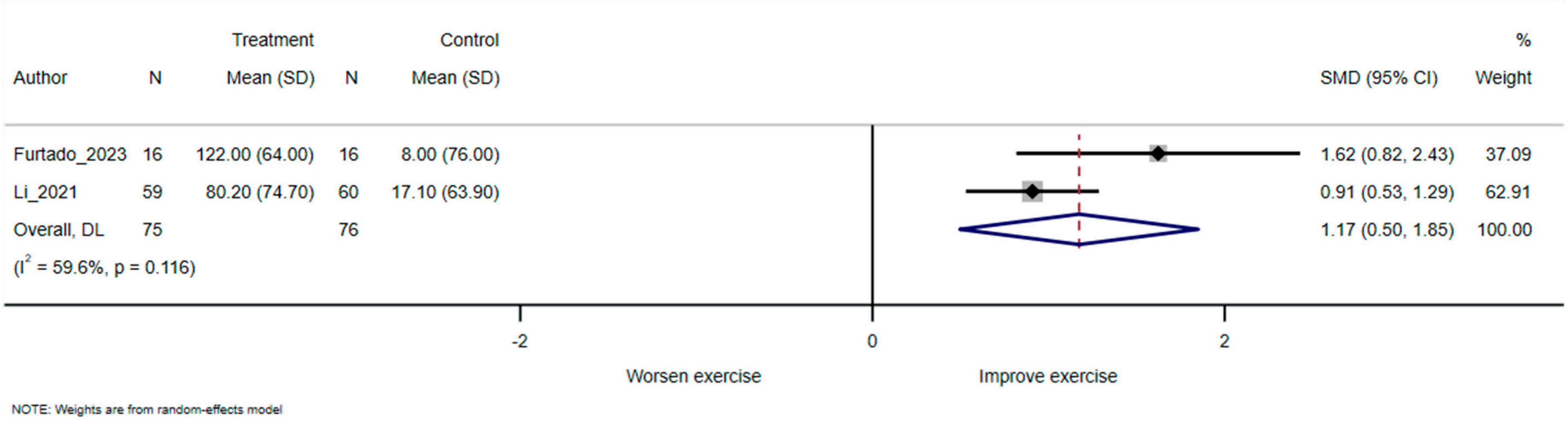

Figure S4a Sensitivity analysis of randomized controlled trials for 6MWD after omitting Amaral et al., 2022. CI, confidence interval; N, number of participants; SD, standard deviation; SMD, standardized mean difference; 6MWD, six-minute walk distance [39,40].

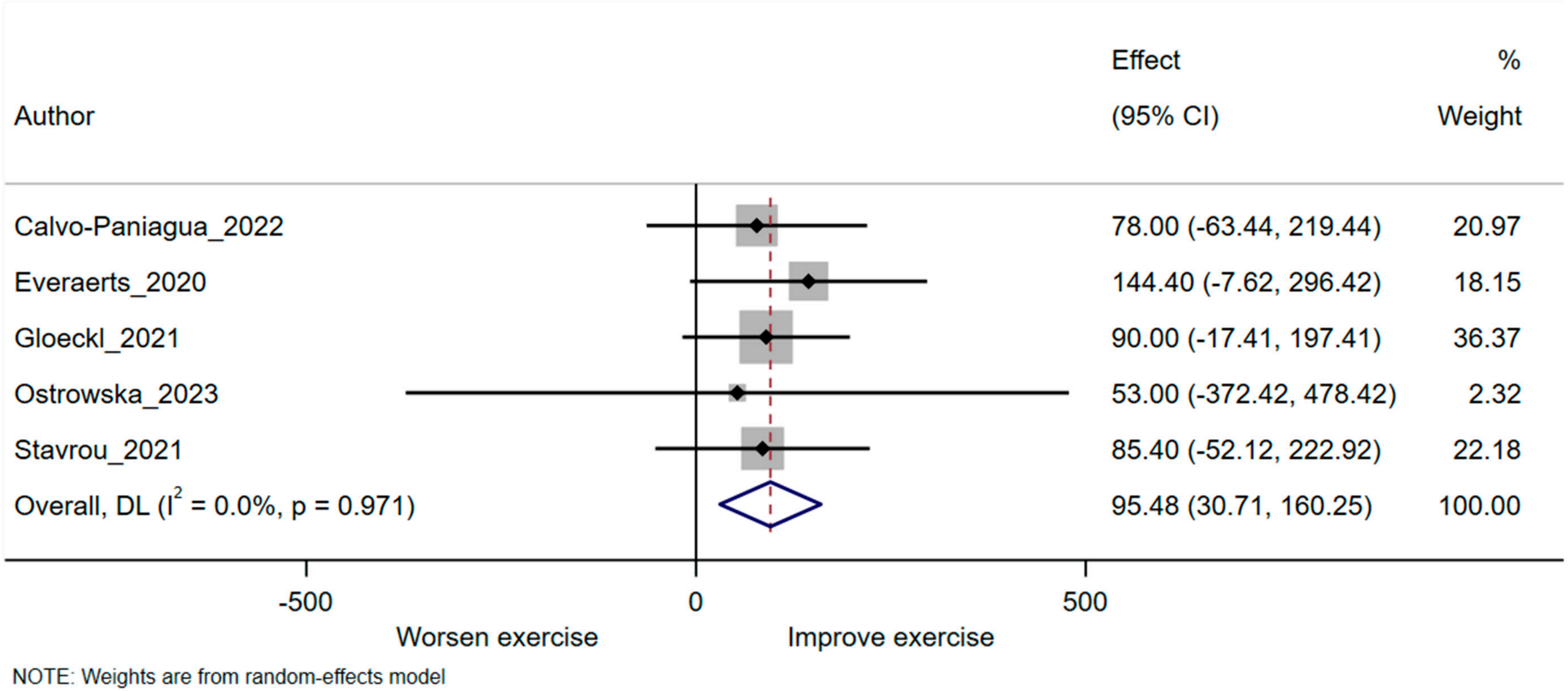

Figure S4b Sensitivity analysis of non-randomized controlled trials for 6MWD after omitting Ahmed et al., 2021. CI, confidence interval; Effect, weighted mean difference; 6MWD, six-minute walk distance [22,42,43,44,45].

Figure S5

a)

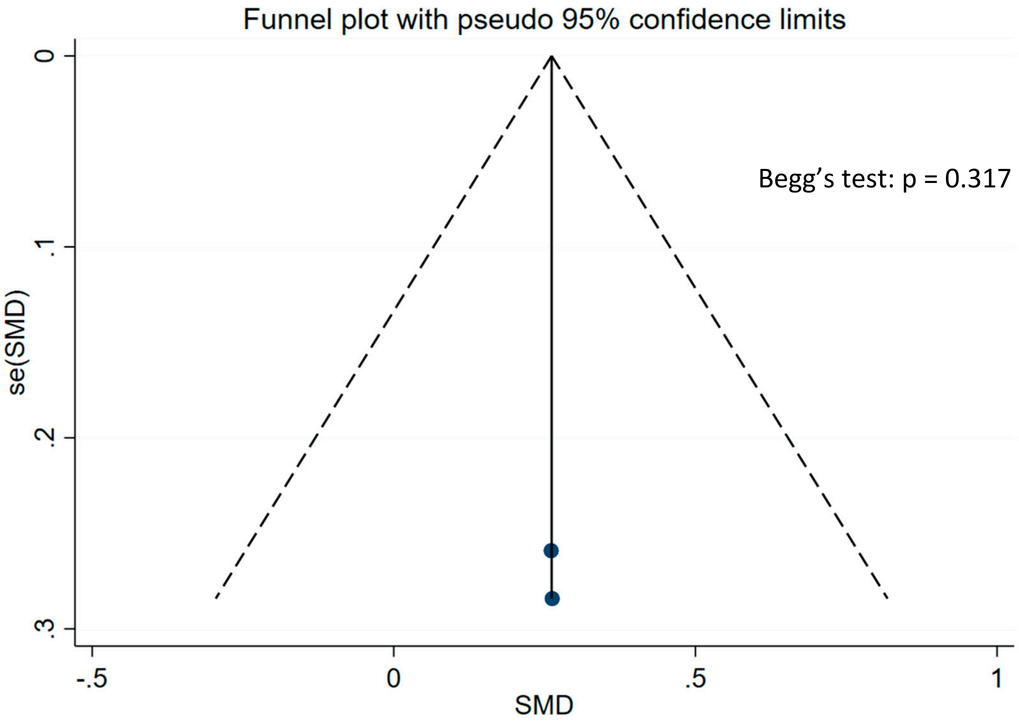
Funnel plot with pseudo 95% confidence limits
Begg's test: p = 0.317
0
.1
.2
.3
se(SMD)
-.5
0
.5
1
SMD

b)

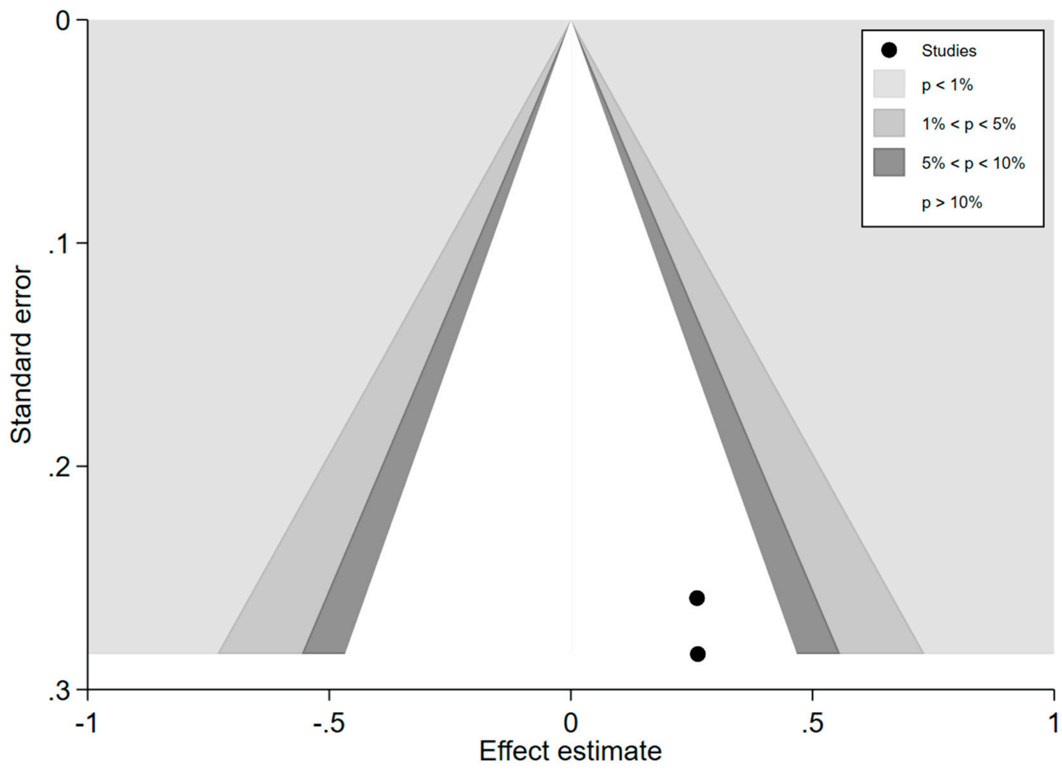

Figure S5. Publication bias of randomized controlled trials for VO_{2peak}. a) Funnel plot and Begg's test; b) Contour-enhanced funnel plot

CI, confidence interval; se, standard error; SMD, standard mean difference; $VO_{2peak,}$ peak oxygen consumption

Figure S6

a)

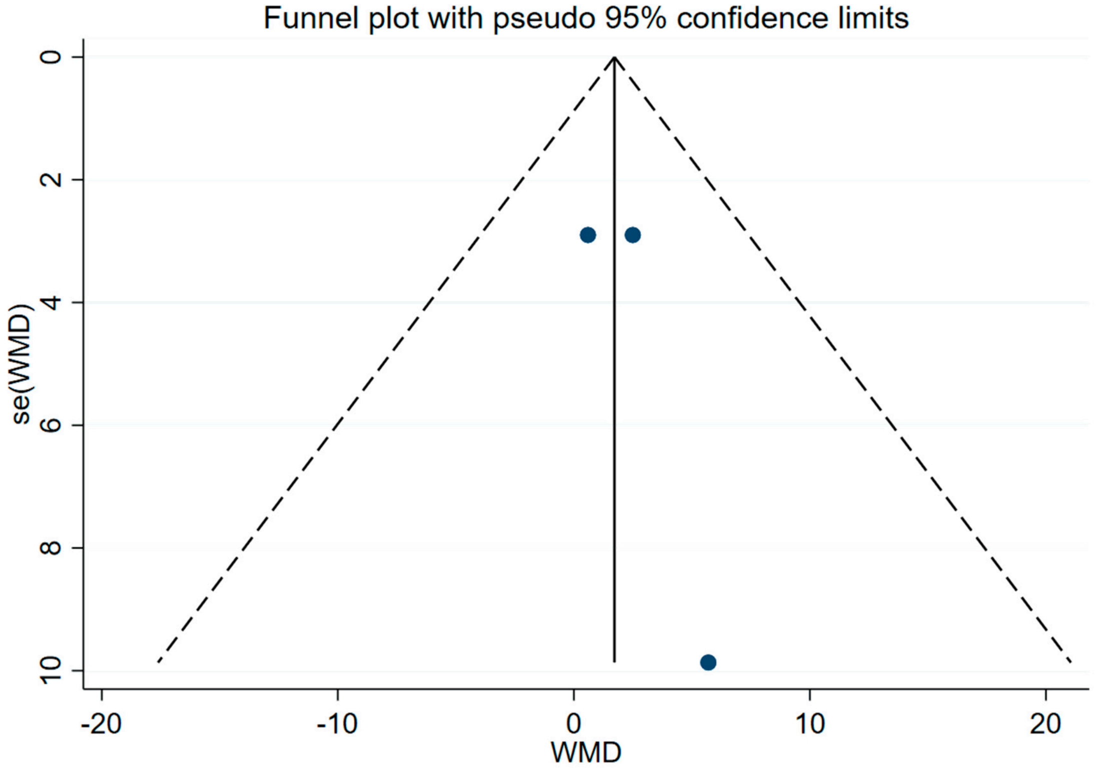

Funnel plot with pseudo 95% confidence limits
0
2
4
6
8
10
se(WMD)
-20
-10
0
10
20
WMD

b)

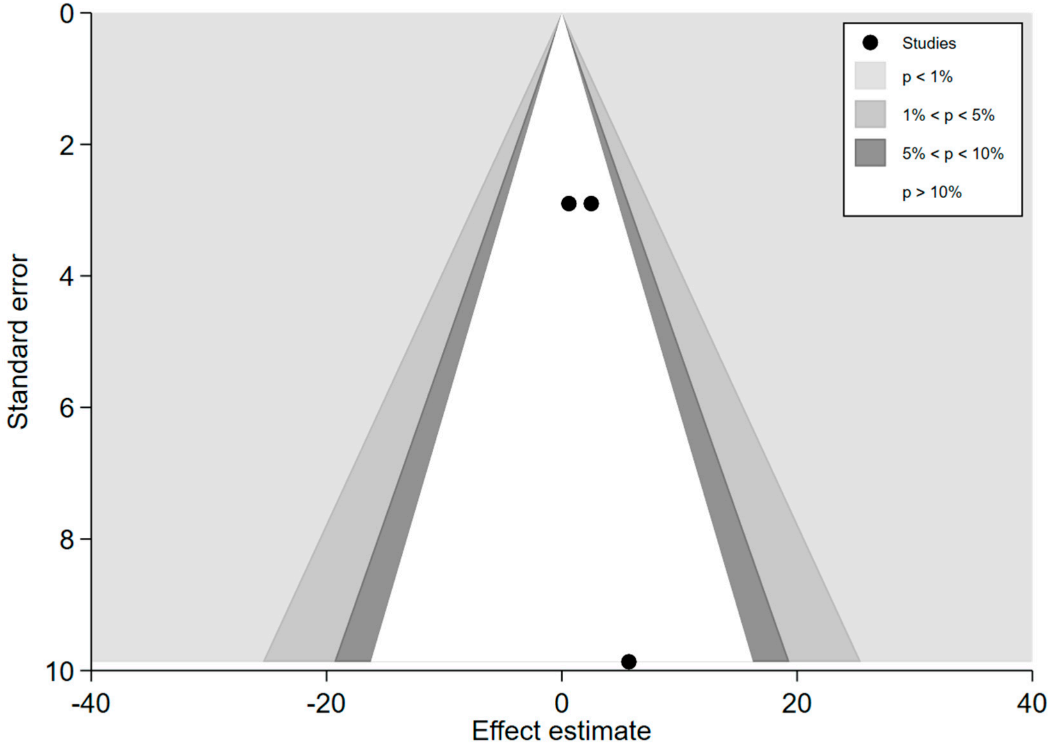

Studies
p < 1%
1% < p < 5%
5% < p < 10%
p > 10%
Standard error
0
2
4
6
8
10
-40
-20
0
20
40
Effect estimate

c)

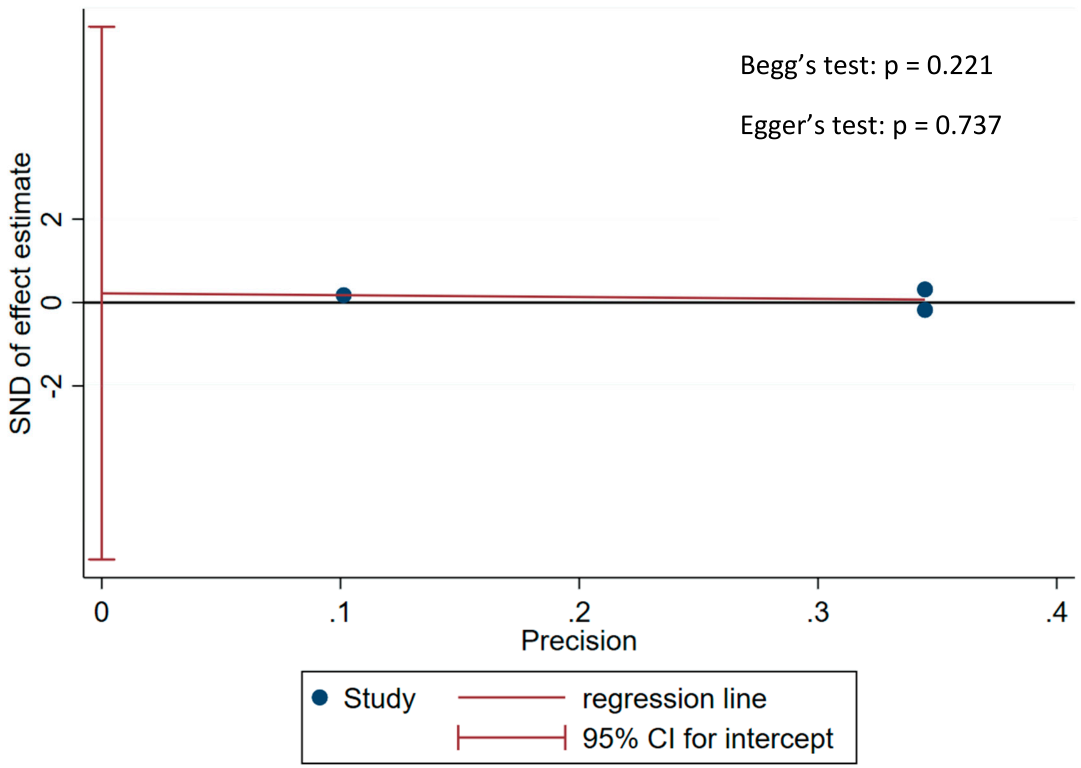

Figure S6. Publication bias of non-randomized controlled trials for VO_{2peak}. a) Funnel plot; b) Contour-enhanced funnel plot; c) Egger's graph and Begg's test

CI, confidence interval; se, standard error; SND, standard normal deviate; WMD, weighted mean difference; VO_{2peak}, peak oxygen consumption

Figure S7

a)

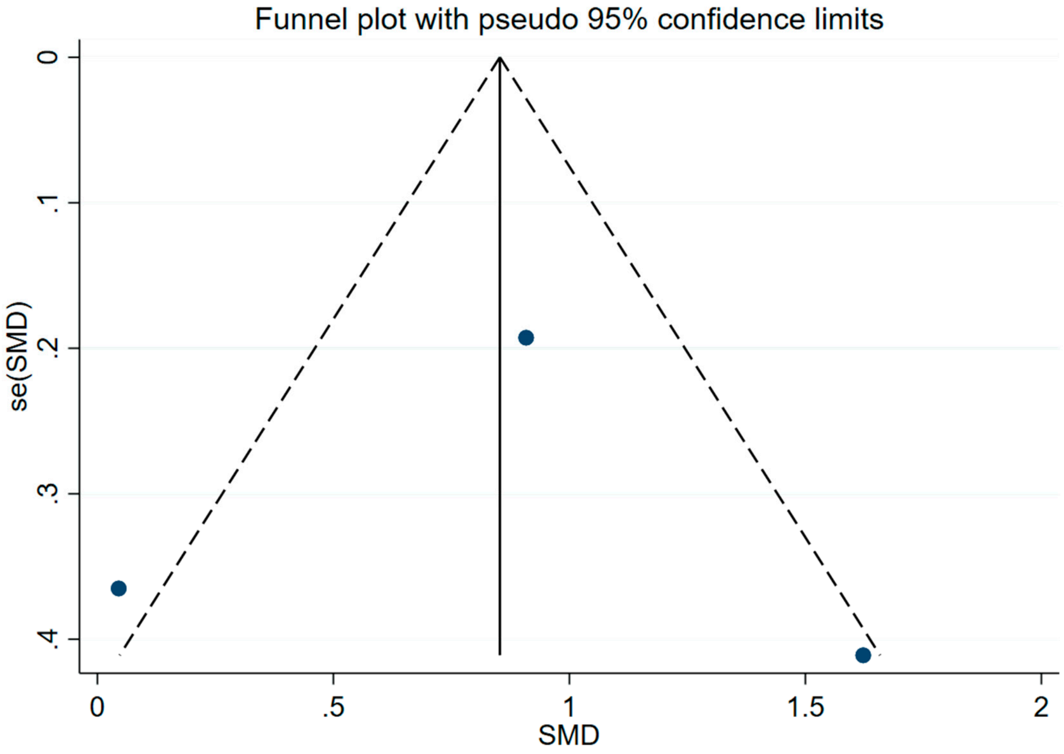
Funnel plot with pseudo 95% confidence limits
se(SMD)
0
.1
.2
.3
.4
0
.5
1
1.5
2
SMD

b)

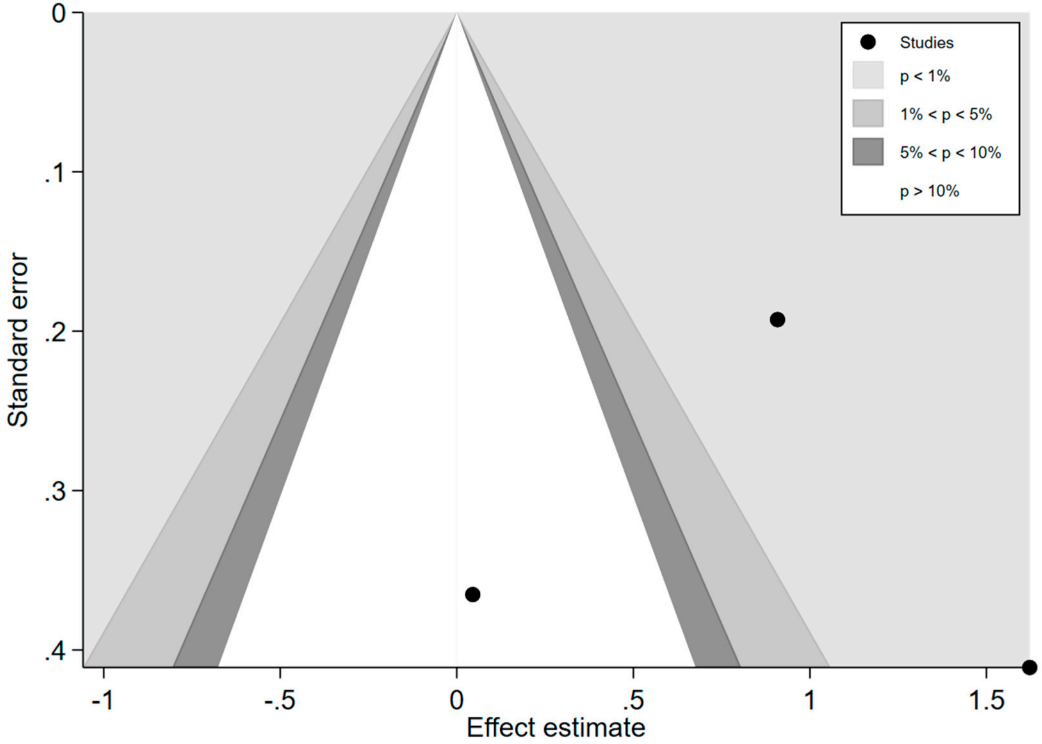

Standard error
0
.1
.2
.3
.4
-1
-.5
0
.5
1
1.5
Effect estimate
Studies
p < 1%
1% < p < 5%
5% < p < 10%
p > 10%

c)

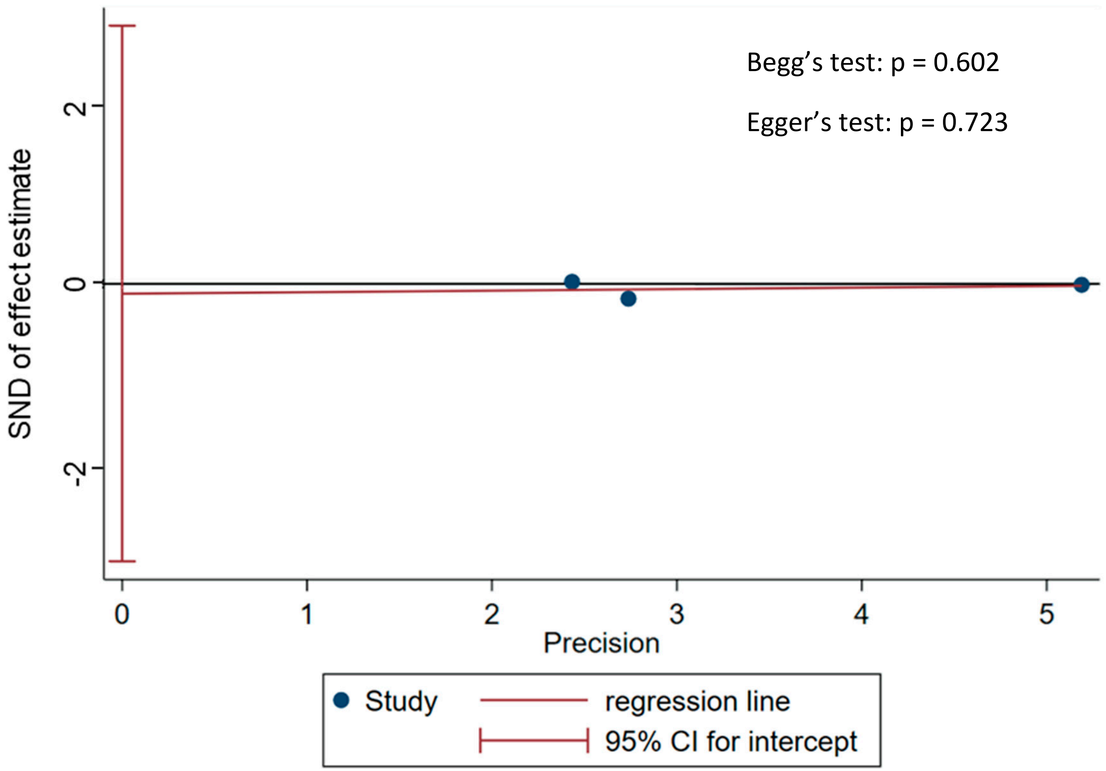

Figure S7. Publication bias of randomized controlled trials for 6MWD. a) Funnel plot; b) Contour-enhanced funnel plot; c) Egger's graph and Begg's test

CI, confidence interval; se, standard error; SMD, standardized mean difference; SND, standard normal deviate; 6MWD, six-minute walk distance

Figure S8

a)

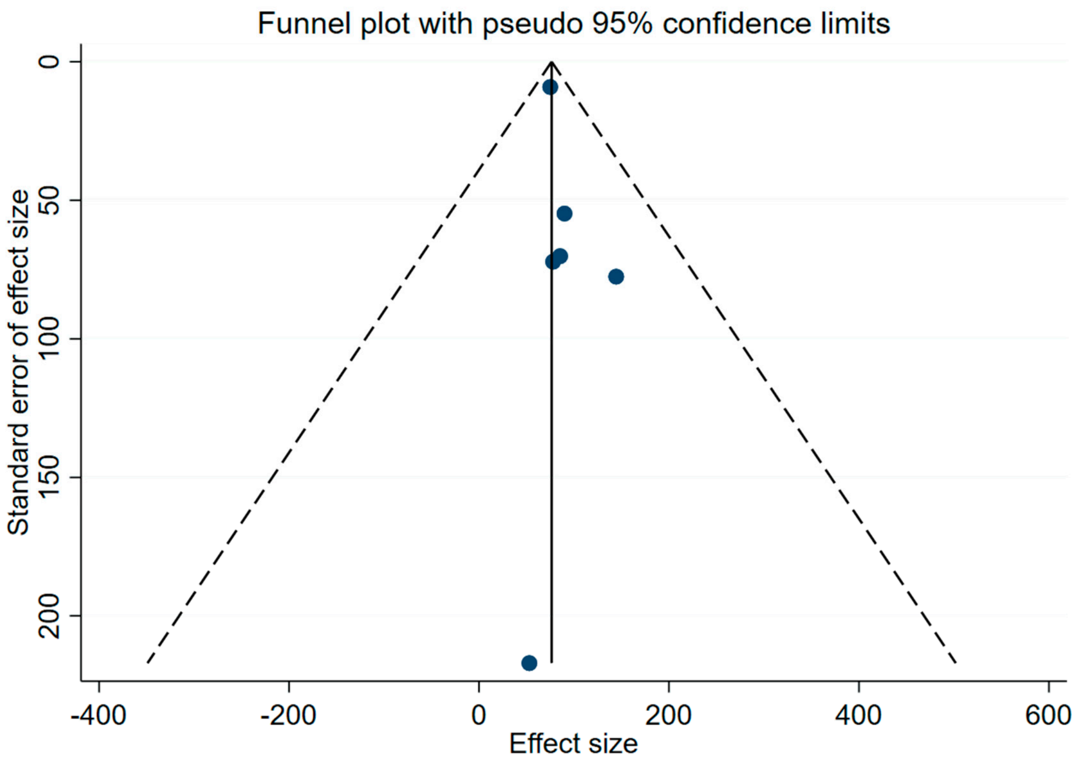

Funnel plot with pseudo 95% confidence limits
0
50
100
150
200
Standard error of effect size
-400
-200
0
200
400
600
Effect size

b)

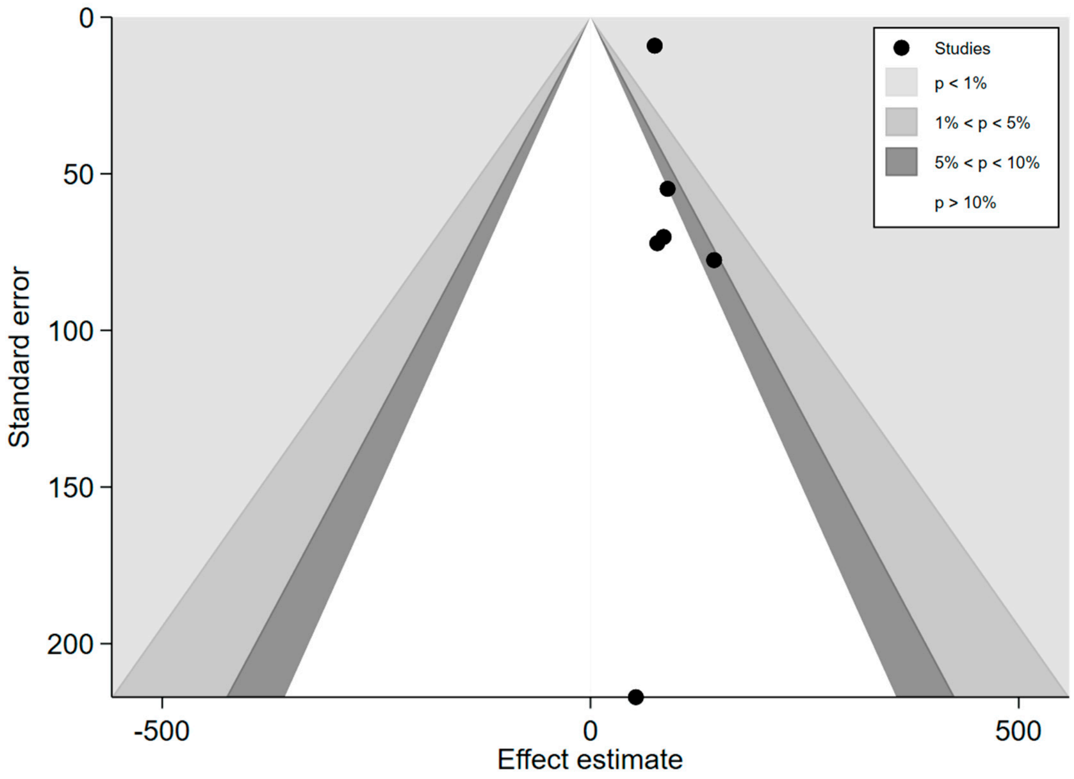

Standard error
0
50
100
150
200
-500
0
500
Effect estimate
Studies
p < 1%
1% < p < 5%
5% < p < 10%
p > 10%

c)

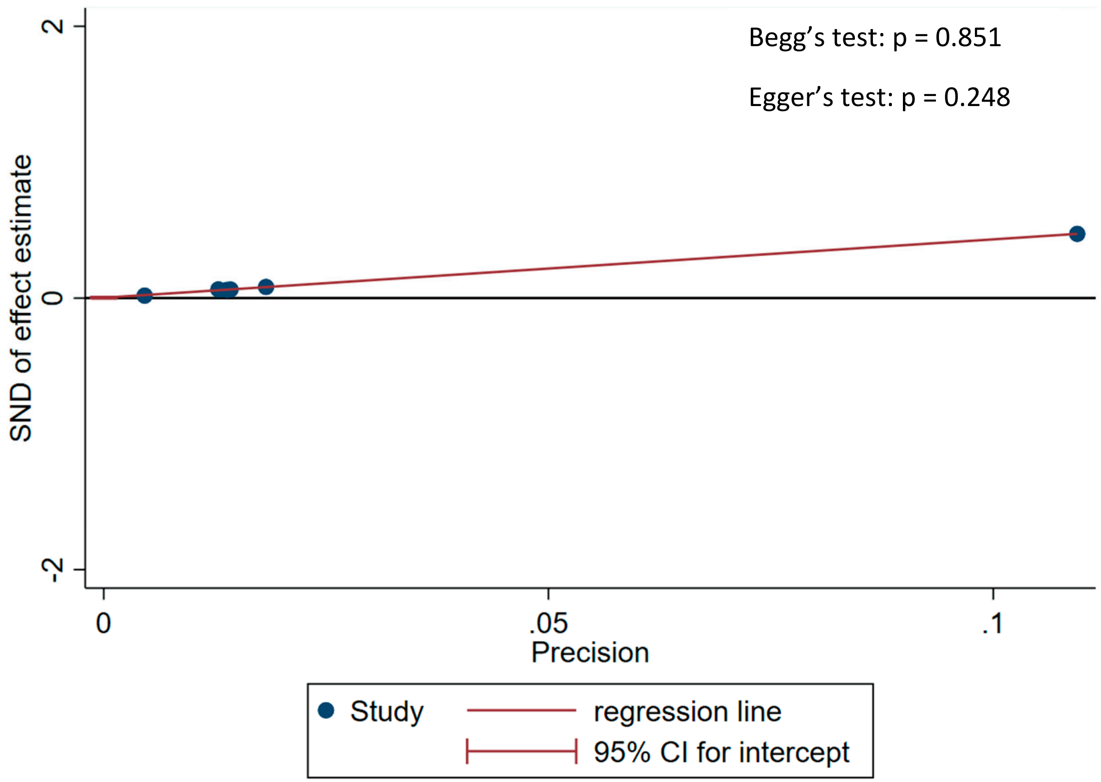

Figure S8. Publication bias of randomized controlled trials for 6MWD. a) Funnel plot; b) Contour-enhanced funnel plot; c) Egger's graph and Begg's test

CI, confidence interval; se, standard error; SMD, standardized mean difference; SND, standard normal deviate; 6MWD, six-minute walk distance
